# Gel with silver and ultrasmall iron oxide nanoparticles produced with *Amanita muscaria* extract: physicochemical characterization, microstructure analysis and anticancer properties

**DOI:** 10.1038/s41598-018-31686-x

**Published:** 2018-09-05

**Authors:** Olena Ivashchenko, Łucja Przysiecka, Barbara Peplińska, Marcin Jarek, Emerson Coy, Stefan Jurga

**Affiliations:** NanoBioMedical Centre, Adam Mickiewicz University in Poznań, 61614 Poznań, Poland

## Abstract

Combination therapy remains one of the most promising and intensively developed direction in cancer treatment. This study is aimed to combine and investigate the anticancer properties of silver nanoparticles (NPs) and *Amanita muscaria* mushroom in gel formulation. For this, hyaluronic acid was used as gel-forming agent, whereas *Amanita muscaria* extract was used as capping agent during silver and ultrasmall iron oxide (MAg) NPs synthesis. *Amanita muscaria* compounds formed NP’s surface layer and contributed anticancer properties, whereas silver NPs contributed anticancer, fluorescence and photoactive properties to the gel. Physicochemical characterization included X-ray diffraction (XRD), microscopies (SEM, cryo-SEM, TEM, confocal fluorescence), spectrofluorometric method, thermogravimetric analysis (TGA), dynamic light scattering (DLS) techniques, energy dispersive (EDS), Fourier transform infrared (FTIR) and ultraviolet–visible (UV-Vis) spectroscopies, zeta-potential and rheological measurements. Microstructure analysis of hyaluronic acid/MAg NPs gel was performed by cryo-SEM technique. We showed that hyaluronic acid is a perfect gel-forming agent from both biomedical and technological points of view. It is well-mixed with MAg NPs forming stable gel formulation; high homogeneity of hyaluronic acid/MAg NPs gel was shown by SEM EDS elemental mapping. Microstructure of the gel was found to be highly ordered and consisted of domains from perforated parallel tubular structures. This finding expanded our understanding of gels and broke the stereotype of gel structure as chaotic network of fibers. Cytotoxicity studies performed on 2D and 3D HeLa cell cultures pointed to a high potential of hyaluronic acid/MAg NPs gel for local treatment of cancer. Cell response was found to be significantly different for 2D and 3D cell cultures that was related to their different cytoarhitecture and gene expression. Thus, the results of the cellular spheroids viability showed that they were significantly more resistant to the cytotoxic action of MAg NPs and their gel formulation than 2D cell culture. Hyaluronic acid used as gelling agent in gel formulation was found to increase an effectiveness of active components (MAg NPs, *Amanita muscaria* extract) probably improving their transport inside HeLa spheroids.

## Introduction

In the past decade, progress has been made in cancer therapy including the discovering of new chemotherapeutic agents, steps in immunotherapy, development of targeted and combination therapies^1,2^. One of the biggest challenges in cancer treatment today is to overcome cancer therapy resistance. Combination therapy, including “pharmaceutical” combination therapy, remains one of the most intensively developed direction to solve this problem.

Silver nanoparticles (NPs), besides their well-known antimicrobial activity, possess also anticancer properties and may potentially improve therapeutic results due to its ability to enhance optical signals^3,4^ (e.g., photodynamic therapy). The coupling of silver with magnetic iron oxide NPs allows to obtain nanomaterials with combined properties of its both components: magnetic, optical and bioactive. Magnetic iron oxides in the form of nanoparticles (magnetite, maghemite), due to their chemical stability and biocompatibility are widely studied for their potential use in biomedical application, namely, magnetic targeting and magnetic resonance imaging^5–7^. Their synthesis procedures have been well studied enabling to produce NPs with tunable size and morphologies. However, their ability to self-organization into highly ordered microstructure as well as its functional properties have not been fully investigated. In our previous work we found the existence of highly ordered microstructure in hydrocolloids of ultrasmall iron oxide NPs (with and without silver NPs) produced with *Ginger rhizome* extract^8^. Herein, the role of ultrasmall iron oxides (USIO) NPs is not to provide magnetic properties suitable for magnetic targeting (they are superparamagnetic) but mainly to provide highly ordered microstructure/matrix to hydrocolloid in which silver NPs are regularly distributed^9^. In addition, they may improve the contrast in magnetic resonance imaging technique, working as a T2 contrast agent^8^. Therefore silver and ultrasmall iron oxides (MAg) nanoparticles hydrocolloids are expected to exhibit high potential for biomedical application, for example, as a multimodal material for cancer therapy.

Today, as a part of the “green” technologies, plant and fruits extracts have been used for NPs synthesis^10^. Their main role in synthesis is to substitute toxic substances in order to reduce the environmental waste and impact. The numerous results demonstrated the advantages of the extracts usage: they can be simultaneously capping (confining NPs size), stabilizing, reducing agent, and, depending of extracts medicinal properties, provide biological activity^11,12^. Besides plants and fruits, macrofungi can be the other object of interest for green nanotechnology and cancer research^13^. Though their medicinal properties are known from ancient time, they are now being used mainly by folk medicines for cancer treatment as well^14^. The ability of mushrooms to inhibit tumor growth is supposed to be mostly by stimulating the immune system, particularly the innate branch, such as macrophages and natural killer cells, but also T cells and their cytokine production^15^. Numerous macrofungal compounds have been shown to possess immunomodulatory activity, including polysaccharides (mostly β- and some α-glucans), proteoglycans, proteins, and various constituents of small molecular mass^15^. Among the mushrooms, *Amanita muscaria* is one of the most interesting. Known as a toxic mushroom, it contains muscarine, muscinol and ibotenic acid which are responsible for its psychoactive effects^16^. Psychotropic effect of *Amanita muscaria* is well known from ancient time and used in shamanic practices around the world wherever the mushroom is distributed^17^. It was reported that poisoning caused by *Amanita muscaria* were followed by complete recovery after 24 h without noticeable side-effects or any organ damage^16^. Latest research also showed that polysaccharides extracted from *Amanita muscaria* have been proved to exhibit significant antitumor activity against sarcoma in mice^17^ as well as to reduce the neurogenic pain^18^. From this point of view, *Amanita muscaria* stands as a promising candidate for the development of nanoparticles for anticancer therapy. Here, we focused on development of gel-form of iron oxide and silver NPs with *Amanita muscaria* extract for localized anticancer therapy. The extract of *Amanita muscaria* played also a role of capping agent during the NPs synthesis.

A gel-form of anticancer medicine for *in situ* injection/application promises such advantage as minimal side toxic effect on whole organism. Hyaluronic acid as gel-forming agent was chosen due to its interesting role in tissue metabolism, low toxicity and suitable technological properties. Hyaluronic acid, an ubiquitous carbohydrate polymer, is a major component of skin where it is involved in tissue aging and repair. Its functions include facilitation of cell migration and proliferation. The process of wound healing is attributed to formation of hyaluronic acid-rich network for further growth of granulation tissue matrix^19,20^. On the other hand, due to the exactly same properties, hyaluronic acid may also be involved in the progression of cancer cells. The cells receptors CD44 and RHAMM are considered to be specific for hyaluronic acid; their increased expression were clinically correlated with cancer metastasis^21^. That is why hyaluronic acid, similarly to folic one, has been used as a marker for some cancer types (e.g., breast, gastrointestinal)^22,23^.

In the present study, we investigated the microstructure of hyaluronic acid/MAg NPs gels by cryo-SEM technique. We observed that the microstructure is highly-ordered and consist of domains from perforated parallel tubes, as opposed to commonly used conception of gel structure as a network of chaotically organized fibers^24^. Moreover, the observed similarity between skeletal muscle fibers and hyaluronic acid/MAg NPs gel microstructure allowed as to conclude that the developed gel would be compatible with biological tissue and may provide the required scaffolding for tubular cells growth. Finally, the anticancer properties of MAg NPs and hyaluronic acid/MAg NPs gel as well as the contribution of *Amanita muscaria* extract to these properties, have been studied *in vitro* using 2D and 3D HeLa cells cultures and bioimaging techniques.

## Results and Discussion

### Characterization of USIO and MAg NPs

#### TEM measurements – morphology

**Figure 1:**
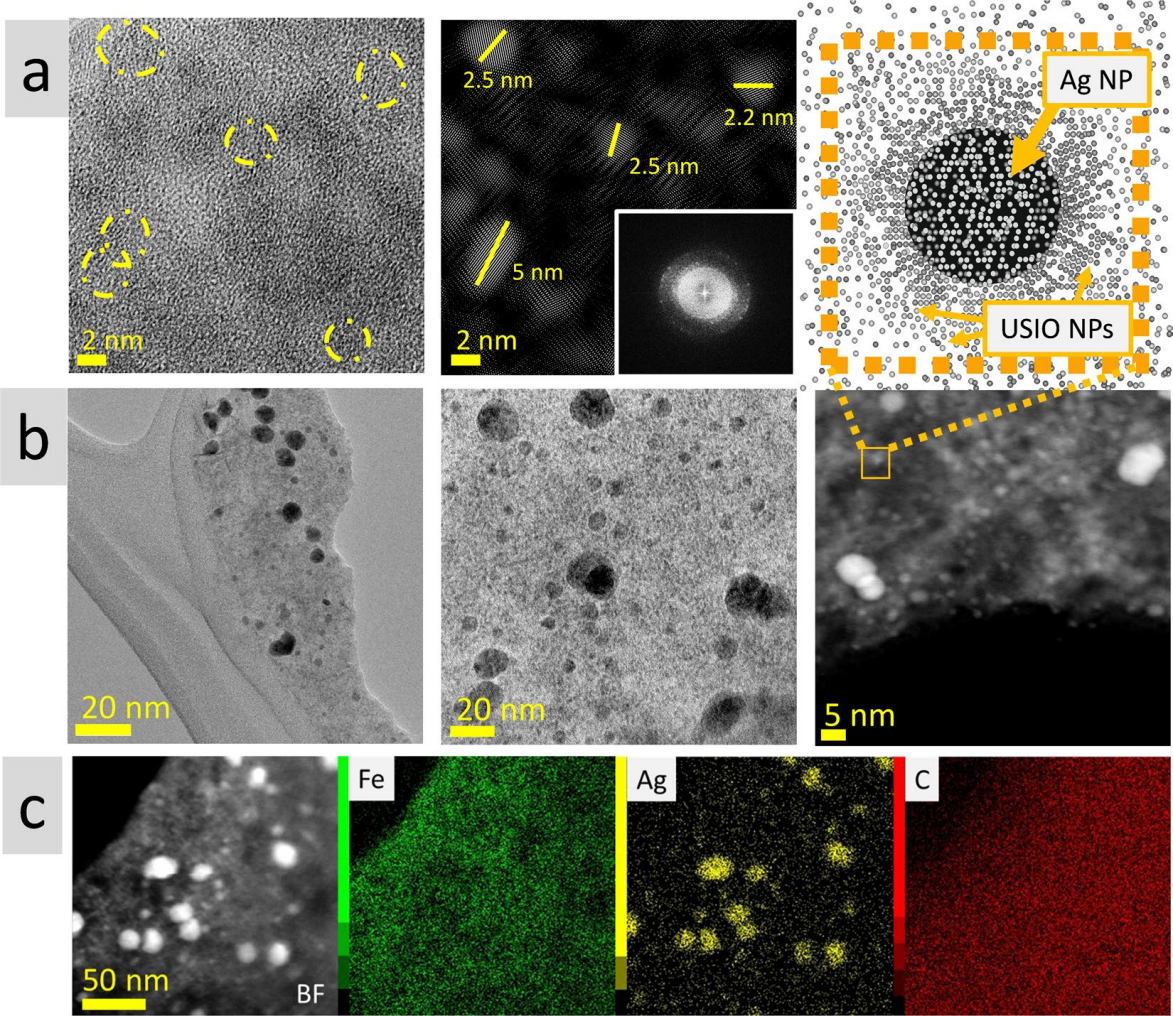
TEM images of. (**a**) USIO NPs (left), FFT analysis (inset) and the result of FFT filtered image using the principal crystalline reflections; (**b**) MAg NPs; (**c**) elements mapping of MAg NPs.

High resolution TEM measurements revealed that USIO NPs are approximately 2 nm in size with narrow size distribution. Post processing of the micrographs using inversed FFT, showed NPs size of 2.2–2.5 nm. Fast Fourier transformation (FFT) analysis showed crystalline nature of USIO NPs (Fig. 1a). TEM measurements of MAg NPs revealed that they consist of two types of NPs: ultrasmall iron oxide and silver. Herein, Ag NPs have 5–25 nm in size and are distributed throughout the ultrasmall iron oxide matrix (Fig. 1b).

#### SEM EDS measurements – elemental analysis

SEM EDS measurements showed that USIO and MAg NPs predominantly consist of iron and oxygen, however, they contain also admixtures originated from *Amanita muscaria* extract used for the synthesis, like carbon (4.7 ± 0.5 wt%), sodium (1.9 ± 0.3 wt%), silicon (1.5 ± 0.4%) and manganese (0.2 ± 0.1%). MAg NPs additionally contain silver (17.5 ± 3.1 wt%) (Fig. S1).

#### XRD analysis – phase composition

**Figure 2:**
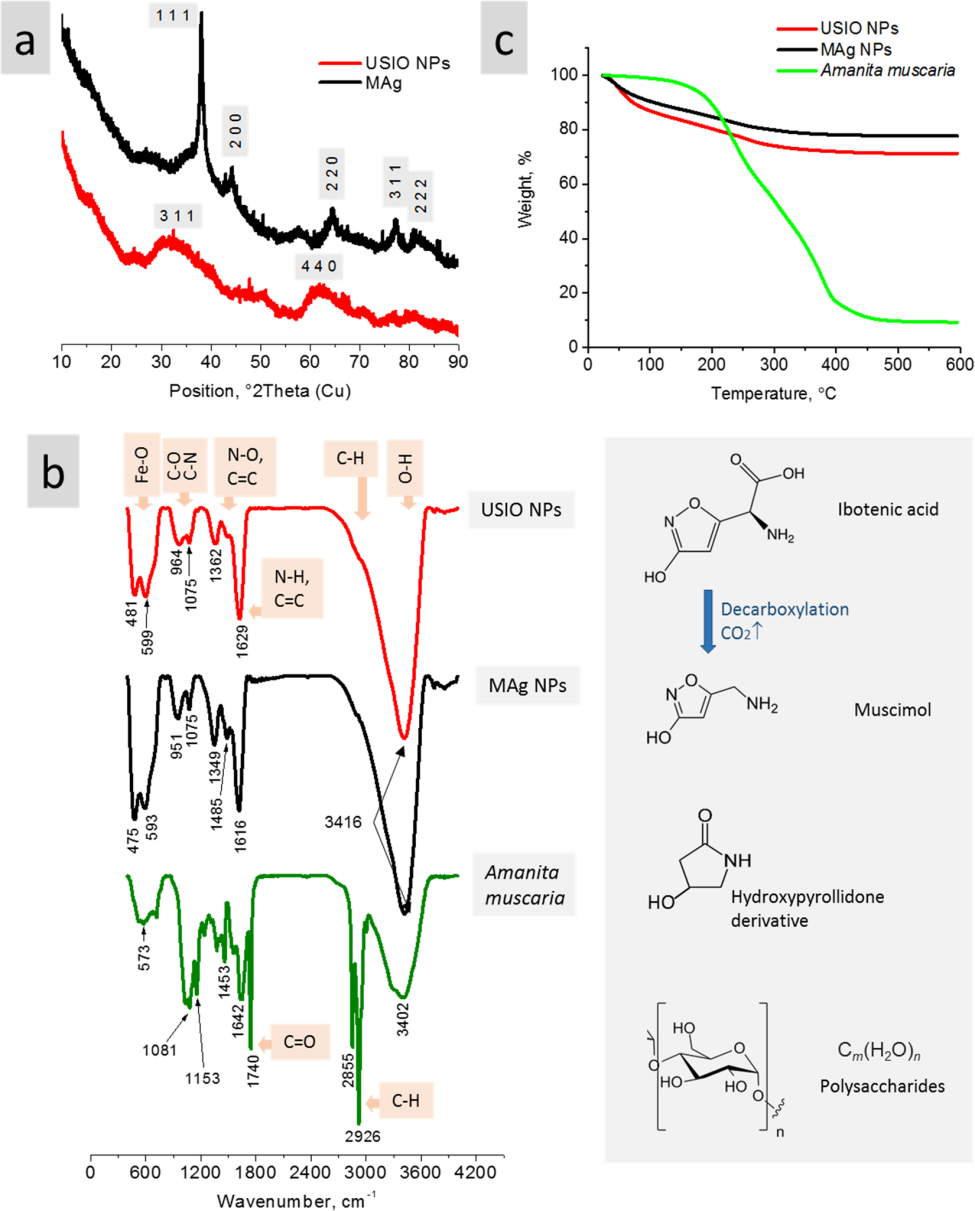
XRD patterns of USIO and MAg NPs (**a**); FTIR spectra of USIO, MAg NPs and *Amanita muscaria* (**b**) and TGA curves for USIO, MAg NPs and *Amanita muscaria*. (**c**) Structural formulas of *Amanita muscaria* compounds: ibotenic acid, muscimol, hydroxypyrollidone derivative, and polysaccharide (**b**, right).

According to the ICDD-PDF4+ database, the peaks were attributed to silver with cubic crystal structure (Ref. code 04-002-3195) (Fig. 2a). Due to the size and associated broadness of the USIO NPs peaks, their precise recognition is intricate; the peaks at ∼35 and ∼63 2Theta are typical to both magnetite and maghemite crystal structure^25^.

#### FTIR spectroscopy measurements – chemical bonds

Even though natural extract is a complicated substance for FTIR spectroscopy analysis, this technique may be very useful for determination of extract compounds adsorbed on NPs surface and involved into their synthesis. Mushroom fruiting bodies consist of approximately 92.5 wt% water, 4.3 wt% carbohydrates, 2.5 wt% proteins, 0.1 wt% fat, 0.6 wt% minerals (K, P, Ca, Mg, Na, Zn, Fe) and 0.006 wt% vitamins (B3, B5, B2, B6, B1)^26^. Besides them, *Amanita muscaria* contains also ibotenic acid, muscimol, hydroxypyrollidone derivatives (structural formulas on Fig. 2b, right)^16,27,28^. Carbohydrates are mainly represented by glucans - polysaccharide that widely distributed in the fungal cell walls; fungi proteins exist in compounds like proteoglycans or glycoproteins where they are covalently bonded with glucans.

The spectrum of *Amanita muscaria* contains bands of Metal‒O bonds (K, P, Ca, Mg, Na, Zn, Fe) (500–600 cm^−1^), C‒O, C‒N (1081&1153 cm^−1^), N‒O (1375&1557 cm^−1^), N‒H and C=C (1642 cm^−1^) bonds^29^ (Fig. 2b). Sharp intensive peaks at 1740, 2855 and 2926 cm^−1^ may be related to C=O (the former peak) and C‒H bonds, respectively. Wide peak at 3402 is attributed to hydrogen-bonded O‒H bonds. The ratio between C‒O, C‒N (1081 cm^−1^) and N‒H, C=C (1642 cm^−1^) is approximately 1:1.

The spectra of USIO and MAg NPs contains bands at 475–599 cm^−1^ which are typical for Fe‒O bonds in magnetite and maghemite structures^30^. The other peaks point to the presence of organic compounds originated from *Amanita muscaria* extract that was used in the NPs synthesis. The peak at 1075 cm^−1^ is characteristic for C‒O stretching vibration in C‒O‒C groups of polysaccharides that means the presence of glucans in the surface layer of NPs^26,31^. The difference in the peak intensity (if compare spectrum of *Amanita muscaria* with spectra of USIO and MAg NPs) allows to conclude that *Amanita muscaria* compounds selectively adsorb on NPs surface: the ratio between C‒O (1081 cm^−1^) and N‒H, C=C (1642 cm^−1^) is approximately 1:3. This means that, in spite of the fact that more than half of the *Amanita muscaria* compounds are carbohydrates, the nitrogen-containing compounds are actively adsorbed by NPs. The peak at 1349 (MAg NPs) and 1362 (USIO NPs) cm^−1^ related to C‒N bonds in amines confirms this supposition. As known, nitrogen-containing compounds in *Amanita muscaria* are ibotenic acid, muscimol, hydroxypyrollidone derivatives^16^, and polysaccharide peptides (glycoproteins, proteoglicans)^32^ (see structural formulas on Fig. 2b, right). Herein, the levels of ibotenic acid in *Amanita muscaria* is usually twice as much as muscimol and may be as high as 480 μg/g (0.048 wt%)^32^; it can be easily converted to muscimol *via* decarboxylation. The fact that the peaks at 1740 cm^−1^ (C=O stretching band of the carboxyl groups) disappeared on the USIO and MAg NPs spectra may be explained by predominant adsorption of muscimol rather than ibotenic acid. It is also possible that ibotenic acid converts into muscimol under the synthesis conditions and/or takes part in reduction of silver and iron ions. Next, the peak of C‒H bonds vibrations (2855–2926 cm^−1^) significantly decreased that points to reduced quantity of polysaccharide peptides on the NPs surface (in comparison with *Amanita muscaria* extract). Thus, it is possible that the surface layer of NPs consists of glucans and muscimol.

Further spectra comparison (taking into account also the peaks shape) revealed that the peaks of *Amanita muscaria* spectrum at 1081, 1153 cm^−1^ (C‒O, C‒N bonds) were shifted (Δ84 ± 0.7 cm^−1^) to lower values on the spectra of USIO and MAg NPs. The peak at 1642 cm^−1^ (N‒H, C=C) also shifted to lower values (Δ19 cm^−1^) for USIO and MAg NPs. These shifts pointed to the straining in C‒O, C‒N, N‒H and C=C bonds caused by the strong adsorption forces or covalent bonding of glucans and muscimol to the NPs surface.

#### TGA analysis

TGA analysis was used to estimate content of *Amanita muscaria* compounds on MAg and USIO NPs (Fig. 2c). Extract dry residue of *Amanita muscaria* revealed thermostability up to ∼152 °C (weight loss ≤3.5%); their most intensive decomposition occurred from 180 to 400 °C. This result is in agreement with previously published data: *Amanita muscaria* toxins cannot be removed from fruiting body while cooking - decomposition of ibotenic acid and muscimol occurs at 150 °C and 175 °C, respectively, whereas muscazon decomposes at 190 °C^33^. *Amanita muscaria* residual sample weight after heating to 600 °C was 9.2%. For USIO and MAg NPs, the initial weight loss range from 35 °C to 80 °C can be attributed to the loss of the moisture. Subsequently, the NPs weight decreased with the rise of temperature up to ∼400 °C, then stabilized giving the final residual weight of 71.4 and 77.6% at 600 °C for USIO and MAg NPs, respectively. According to the remaining weight of NPs and *Amanita muscaria* dry residue, the content of *Amanita muscaria* compounds on USIO and MAg NPs was calculated to be 31.5 and 24.7 wt%, respectively.

#### UV-Vis spectroscopy

*Amanita muscaria* spectrum revealed a peak at approximately 255 nm, USIO NPs absorb light from ∼430 to 200 nm. MAg NPs spectrum has two overlapped peaks at 350 and 430 nm that are assigned to the surface plasmon resonance effect of Ag NPs^34^ (Supplementary Fig. S1c).

#### DLS measurements – agglomerates size distribution

Number weighted results of DLS measurements of USIO NPs revealed that in aqueous dispersions (OD ≤1) they exist as agglomerates with size approximately 16 nm at room temperature (22 °C), and with size 83 nm at 37 °C. MAg NPs in aqueous dispersions behaved in similar way: their agglomerates were approximately 16 nm in size at room temperature, and 71 nm at 37 °C (Supplementary Fig. S2a).

#### Zeta-potential measurements – surface charge

Mean zeta-potential of USIO NPs in aqueous dispersions was −34.3 ± 0.9 mV at room temperature, and remained the same at 37 °C (−34.5 ± 1.1 mV). Similar results were obtained for MAg NPs: −37.6 ± 1.4 mV at room temperature, and −34.7 ± 2.1 mV at 37 °C (Supplementary Fig. S2b).

#### Fluorescence measurements

**Figure 3:**
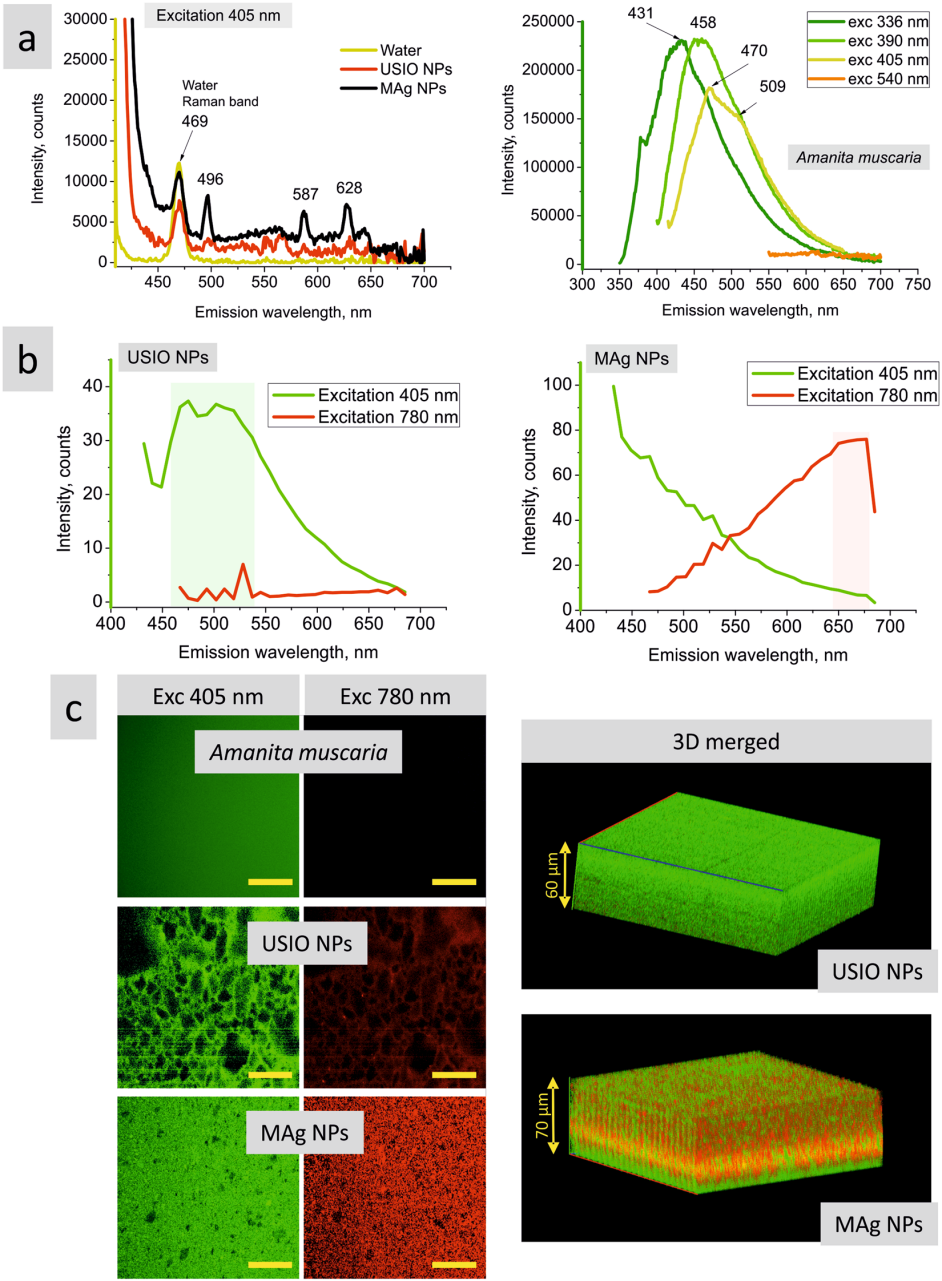
Fluorescence emission spectra of USIO and MAg NPs aqueous dispersions and *Amanita muscaria* extract (**a**) (OD < 1). Fluorescence emission spectra (**b**) and images (**c**) of USIO and MAg NPs hydrocolloids (37.1 ± 1.1 mg/ml). Scale bar corresponds to 50 µm.

Fluorescence emission spectrum of USIO NPs diluted aqueous dispersion (OD ≤1) at excitation wavelength 405 nm revealed, besides water Raman band, weak peaks closely to the level of noise (Fig. 3a). MAg NPs spectrum revealed the narrow peaks at 496, 587 and 628 nm. Taking into account the shape of these peaks and their shift at excitation wavelength of 390 nm (Supplementary Fig. S3a), they were assigned to a Raman effect (probably surface-enhanced due to the presence of silver)^8^. In contrast to the diluted aqueous dispersions, USIO and MAg NPs in hydrocolloids release fluorescent emittance (Fig. 3b,c). As seen, USIO NPs emit fluorescence at excitation wavelength 405 nm, whereas MAg NPs – at 405 and 780 nm. As *Amanita muscaria* extract revealed fluorescence emission at excitation wavelengths within 336‒405 nm (Fig. 3a and S3b), it is reasonable to assume that fluorescence emission of USIO NPs is originated from *Amanita muscaria* compounds, whereas that of MAg NPs is due to both surface plasmon resonance effect of silver NPs and *Amanita muscaria* compounds. 3D fluorescence image of MAg NPs demonstrates the uniformity of Ag NPs distribution in ultrasmall iron oxide matrix (Fig. 3c).

### Characterization of hyaluronic acid/MAg NPs gel

The idea of creation of MAg NPs in gel form was to achieve prolong local therapeutic effect after *in situ* injection or application; from this point, the slower solubility in biological media the longer therapeutic activity. To find suitable basis of gel, several biocompatible polymers (poly(vinylpyrrolidone), poly(caprolactone), poly(vinylacetate), gelatin from bovine skin and cold fish, agar and agarose, and hyaluronic acid) were tested as gel-forming agents for preparation of gel with MAg NPs. It was found that, from technological point of view (miscibility and injectibility), gelatin and hyaluronic acid were the most suitable polymers. Among them, hyaluronic acid was finally chosen due to its interesting role in tissue metabolism^15^.

#### Gel viscosity, solubility in water, PBS and BSA solutions

**Figure 4:**
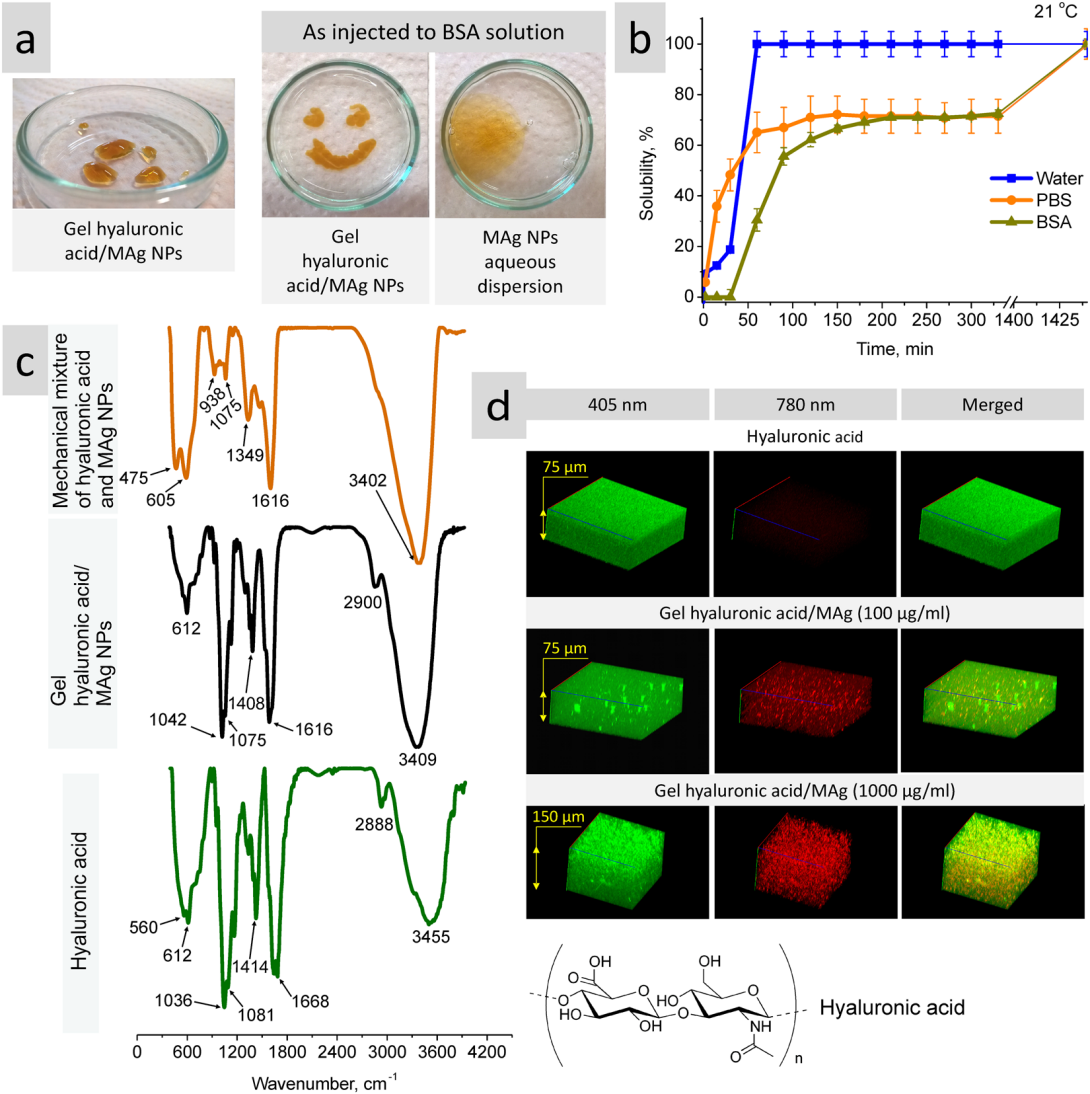
Photos of hyaluronic acid/MAg NPs gel (MAg NPs concentration in gel was 1 mg/ml) on air (left) and just injected to BSA solution (right) in comparison with MAg NPs aqueous dispersion with the same concentration (**a**); kinetic of dissolution of hyaluronic acid/MAg NPs gel (MAg NPs concentration in gel was 1 mg/ml) in water, PBS and BSA solutions at 21 °C (**b**); FTIR spectra of hyaluronic acid, gel hyaluronic acis/MAg NPs and mechanical mixture of hyaluronic acid and MAg NPs (1:1) (**c**) and fluorescence 3D images of hyaluronic acid and hyaluronic acid/MAg NPs gels (**d**).

Hyaluronic acid/MAg NPs gel is a transparent yellow viscous liquid (Fig. 4, left); its viscosity is suitable for injection. Rheological measurements (shear rate *vs* shear viscosity) revealed (i) non Newtonian character of gels and (ii) that addition of MAg NPs caused decrease of gel viscosity (on ∼12%, from 3228 to 2843 mPa·s) (Supplementary Fig. S4c). MAg NPs in gel form keep their consistence after injection to BSA solution in contrast to MAg NPs aqueous dispersion (see photos on Fig. 4, right). The study of kinetics of solubility of hyaluronic acid/MAg NPs gel showed that the fastest solubility was in water (∼60 min), whereas in PBS and BSA solutions the gel dissolution was significantly slower (>5 h) (Figs 4b and S4).

#### FTIR measurements of hyaluronic acid, hyaluronic acid/MAg NPs gel and mechanical mixture of hyaluronic acid and MAg NPs

FTIR measurements of hyaluronic acid/MAg NPs gel in comparison with its components (MAg NPs (see Fig. 2b) and hyaluronic acid) and their mechanical mixture would give an information about the nature of interaction between gel components, namely, nanoparticles and hyaluronic acid (Fig. 4c).

The spectrum of hyaluronic acid revealed triple intensive peak at 1036&1081&1153 cm^−1^ (C‒O and C‒N stretching vibrations), the peaks at 1414 and 2888 cm^−1^ (C‒H bonds stretching and bending vibrations), and intensive double peak at 1623&1668 cm^−1^ (N‒H bending vibration). The strong broad band at 3455 cm^−1^ was assigned to hydrogen-bonded O‒H groups. An intensive double peak at 560&612 cm^−1^ was related to Na‒O bonds of hyaluronic acid sodium salt used for measurements.

The spectrum of hyaluronic acid/MAg NPs gel mimic the spectrum of hyaluronic acid, however, all the peaks are shifted: the peak at 1036 to 1042 (Δ6 cm^−1^), 1081 to 1075 (Δ6 cm^−1^), 1414 to 1408 (Δ6 cm^−1^), 1623 to 1616 (Δ7 cm^−1^), 2888 to 2900 (Δ12 cm^−1^), and 3455 to 3409 (Δ46 cm^−1^) cm^−1^. This result pointed to hyaluronic acid involvement into interaction (with NPs) based on electrostatic and H-bonds forces.

In contrast to hyaluronic acid/MAg NPs gel spectrum, the spectrum of mechanical mixture exhibited peaks which are inherent to MAg NPs (see Fig. 2b) and completely overlap the bands of hyaluronic acid. This observation pointed to the fact that MAg NPs admixture (not bonded) in hyaluronic acid gives an intensive FTIR signal without any significant shift. Invisibility of typical for MAg NPs Fe‒O peaks (475&605 cm^−1^) might be related to their complete coating and incorporation into hyaluronic acid structure; the shift of all the typical hyaluronic acid peaks in hyaluronic acid/MAg NPs gel spectrum confirms this supposition and pointed to rather strong adhesive (electrostatic, H-bonding) interaction between them.

#### Fluorescence properties of gels

The 3D fluorescence images of hyaluronic acid and hyaluronic acid/MAg NPs gels demonstrated that (i) hyaluronic acid gel emits fluorescence at excitation wavelength 405 nm, whereas hyaluronic acid/MAg NPs gels emit fluorescence in two regions (at excitation 405 and 780 nm) due to the surface plasmon resonance of silver NPs^35^ (Fig. 4d). And, (ii) quite expectably, the intensity (brightness) of fluorescent emittance increased with increase of MAg NPs concentration in gel; the images also demonstrate uniform distribution of NPs in hyaluronic acid matrix, which is of great importance for future biomedical application.

#### Cryo-SEM measurements – microstructure analysis

Cryo-SEM is a relatively novel technique that allow to observe inner structure of the water-contained samples “as it is”, without drying and vaccum exposure, with minimalized influence on microstructure during sample preparation for measurements. The greatest advantage of this technique is fast freezing of sample during which water freezes in amorphous state.

Highly-ordered microstructure of MAg NPs hydrocolloid was found to be similar to that described earlier^8,9^. Herein, ultrasmall iron oxide NPs form dynamic matrix in which silver NPs are regularly distributed and surrounded by iron oxide NPs; this system is dynamic by nature. The images on supplemental Figure S5 demonstrate highly-ordered parallel stripes and sponge-like structures. Stripes and sponges structures are considered to be the same structure observed from different visual angles. Herein, the stripes were as walls of porous tubes with parallel alignment and the sponge was porous tubes cross-section. Depending on the sample cutting/breaking point, we observed stripes or sponge, the same structure at different cross-section. The interlamellae distance (tubes diameter) was ≤5–8 µm.

In order to clear up an influence of *Amanita muscaria* extract on the microstructure of MAg NPs hydrocolloid, different concentrations of the extract (16.2 and 72.0 mg/ml) were investigated. As seen (Supplementary Fig. S6), the extract exhibited highly ordered microstructure with prolong and spongy elements that are perforated at extract concentration 16.2 mg/ml and dense at 72.0 mg/ml. The prolong and spongy elements are from porous tubes with parallel alignment. Their diameter was ≤8 µm. This result points to a significant influence of *Amanita muscaria* extract on MAg NPs microstructure. Similar effect has been observed for *Ginger rhizome* extract^8^. It seems plausible that, polysaccharides (in our case, from *Amanita muscaria* polysaccharide peptides) are responsible for the formation of such microstructure in nanoparticles hydrocolloid.

Hyaluronic acid gel microstructure consists of tubes with parallel alignment with a diameter of ≤5–8 µm (Supplementary Fig. S7). The peculiarity of hyaluronic acid microstructure is a plurality of fibers with a diameter of <100 nm, that form the above mentioned porous tubes.

**Figure 5:**
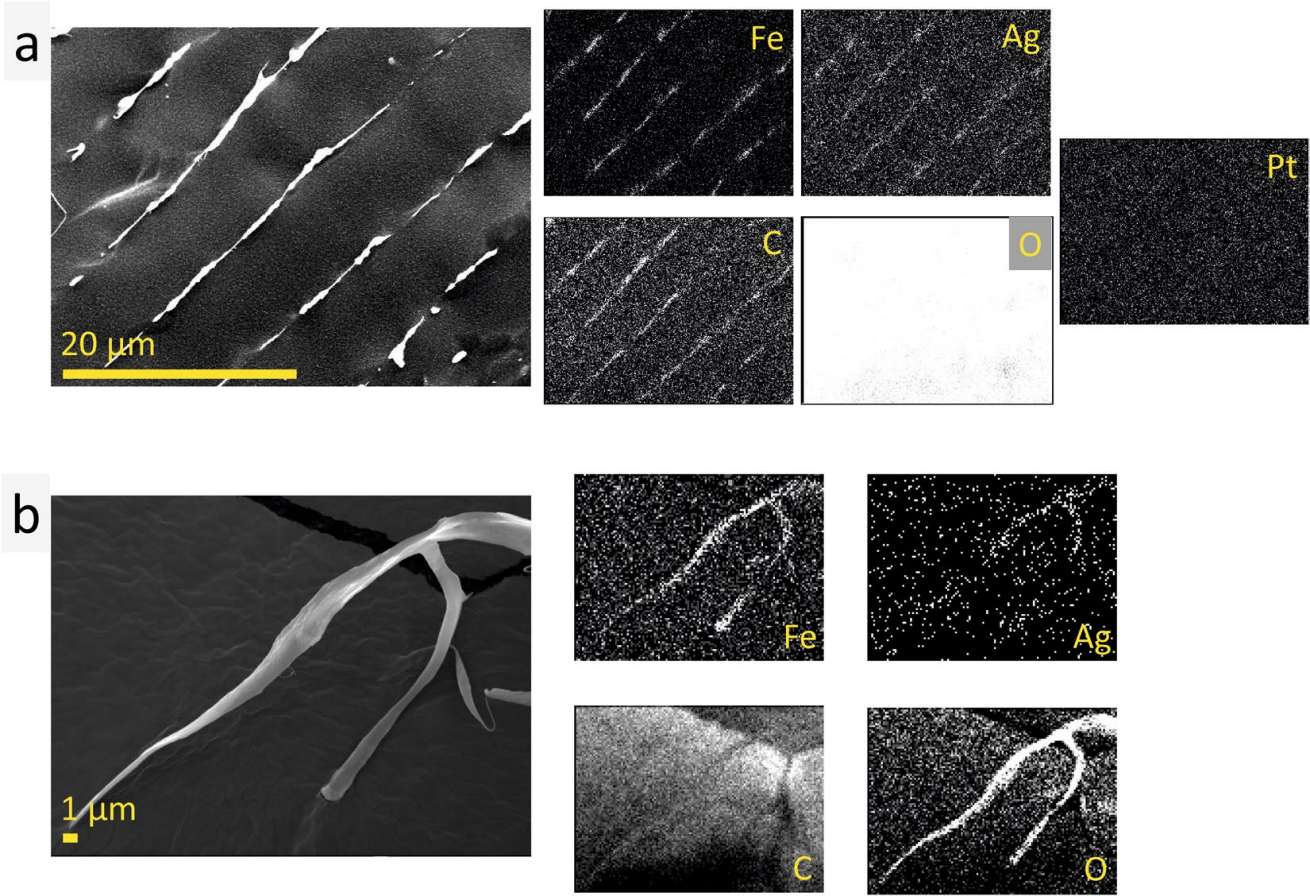
Cryo-SEM EDS elemental analysis of hyaluronic acid/MAg NPs gel (nanoparticles concentration 10 mg/ml) showing maps of Fe, Ag, C and O distribution (note that the amount of oxygen in water is significantly higher than in hyaluronic acid and MAg NPs that is resulted in such an intensive signal); the map of Pt element (used for spattering of sample surface during measurements) is given for comparison (**a**); SEM EDS mapping of freeze-dried hyaluronic acid/MAg NPs gel (nanoparticles concentration 1 mg/ml) showing Fe, Ag, C and O distribution (note that the measurements were performed on carbon tape that resulted in intensive carbon element signal) (**b**).

The microstructure of hyaluronic acid/MAg NPs gel was studied for different NPs concentrations in gel: 50 µg/ml, 100 µg/ml, 1 mg/ml and 10 mg/ml. The use such a significant difference in concentrations was motivated by the supposition that this might have a noticeable effect on the gel microstructure. The measurements revealed that, in general, the microstructure of gels was similar to the one of hyaluronic acid (Supplementary Figs S8 and S9) - porous tubes with parallel alignment (with a diameter of ≤5–8 µm). The fibers, typical feature of hyaluronic acid microstructure, were observed in all the samples studied. One may notice that the increase of NPs concentration in gel to 10 mg/ml improved the microstructure parallel alignment. To be sure of the cryo-SEM results, hyaluronic acid/MAg NPs gel was freeze-dried (−85 °C, 4 days). SEM images of the freeze-dried gel showed very similar to cryo-SEM microstructure and additionally revealed the existence of large areas (>1 mm) oriented in the same direction (Supplementary Fig. S10). Cryo-SEM and SEM EDS elemental analysis showed that Fe, Ag, C elements are localized in microstructure elements (Fig. 5) that confirm the MAg NPs incorporation into hyaluronic acid microstructure (namely, into fibers). These observations pointed to the high homogeneity of hyaluronic acid/MAg NPs gel: even at the highest NPs concentration they did not agglomerate or separate in gel. The compatibility and similarity of MAg NPs and hyaluronic acid microstructures may be attributed to the fact that both of them contain polysaccharides in their structure: MAg NPs – on the surface (originated from *Amanita muscaria* extract), hyaluronic acid - by its chemical nature.

**Figure 6:**
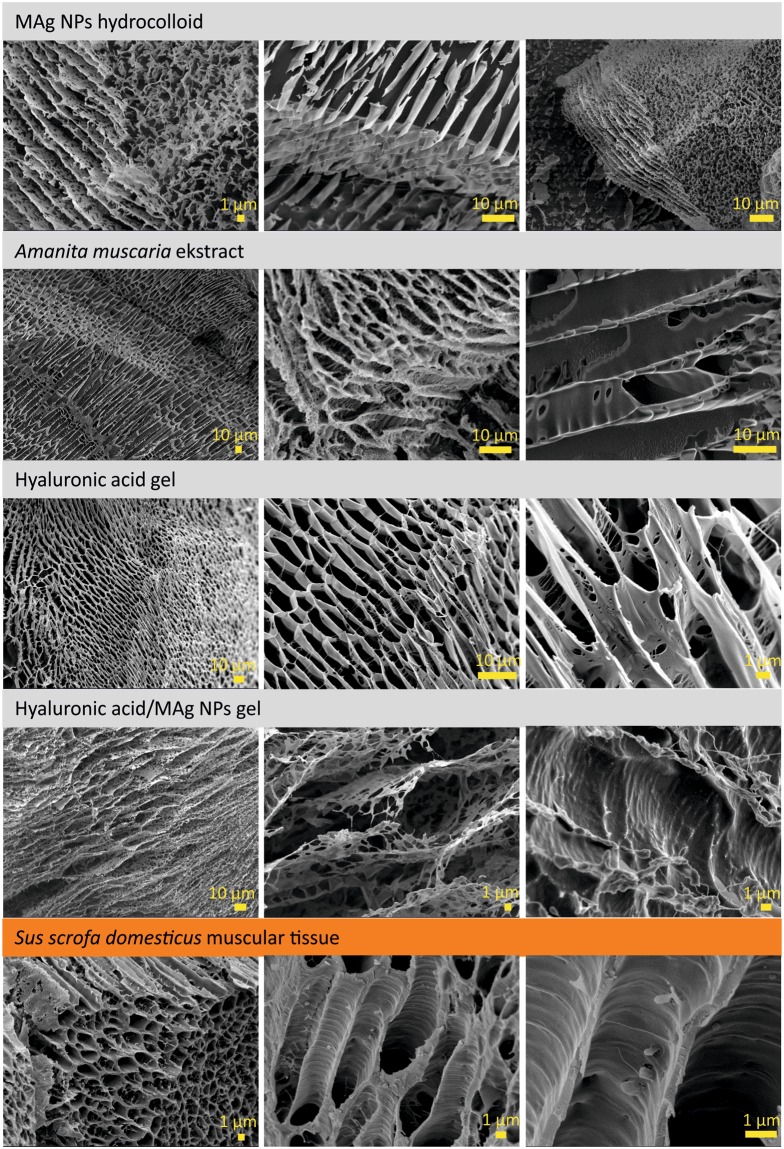
Cryo-SEM images of MAg NPs hydrocolloid (37.1 ± 1.1 mg/ml), *Amanita muscaria* extract (72.0 mg/ml), hyaluronic acid gel (5 mg/ml), hyaluronic acid/MAg NPs gel (nanoparticle concentration 10 mg/ml) and pig *Sus scrofa domesticus* skeletal muscular tissue, which demonstrate similarity of their microstructure.

**Figure 7:**
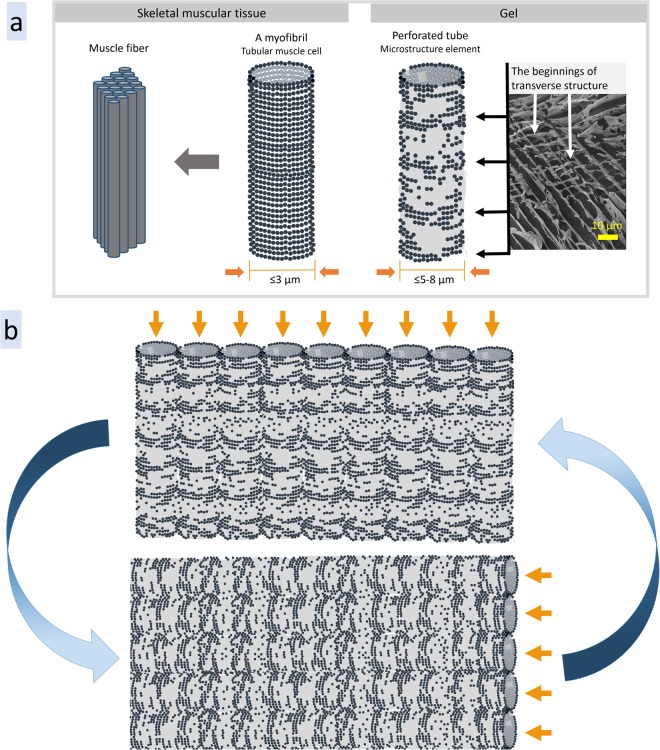
The schemes of: skeletal muscular tissue and gel microstructure element, porous tube, with beginnings of transverse structure (**a**); the principle of microstructure mobility in hydrocolloids and gels - the beginnings of transverse tubular structure become dominant direction in tubes alignment under external forces (e.g., shaking, flowing) (**b**).

The summarized results of cryo-SEM measurements demonstrating the similarity of microstructure of all the samples discussed above are shown on Fig. 6. Interestingly, the same microstructure, porous tubes with parallel alignment, is inherent for skeletal muscular tissue of pig *Sus scrofa domesticus* (see cryo-SEM images on Figs 6 and S11). Pigs are known to be a species closest to humans: the vital organs in pigs and humans share high level of similarity in structure^36^. That is why they have been largely used in medical research^37^. Porous myofibrils, a basic rod-like units of a muscle, are clearly visible on cryo-SEM images. Their diameter is ≤3 µm, smaller than that of tubular structures in hydrogels or hydrocolloids described above, but their walls are significantly denser (Fig. 7a). As cell membranes contain proteoglycans^38^, hybrid protein-polysaccharide molecules, so this similarity may be related to polysaccharides ability to form tubular microstructure in aqueous media.

The observed similarity between muscle fibers and hyaluronic acid/MAg NPs gel microstructure allowed us to conclude that the developed gel would be compatible with biological tissue and may provide the scaffolding for tubular cells growth. This ability of gel microstructure to mimic matrix cells environment has been already utilized for 3D cell culture *ex vivo*^39^. Nowadays, both synthetic and natural hydrogels are used as scaffolds for cell culture growth^40^.

The compatibility of microstructure of muscle fibers and hyaluronic acid/MAg NPs gel may be useful for remodeling of the damaged tissue *in vivo*. As the muscle tubular cells always have fixed direction, so they may determine and force the reorganization of gel tubular structure in a needed direction, due to the gel microstructure mobility. The porous tubes alignment in gels and hydrocolloids is dynamic and may be easily changed by external factors (e.g., shaking, flowing, etc.). This mobility of the microstructure may be explained by existence of transverse tubular structure that plays a role of skeleton (matrix) for changing of tubes direction (Fig. 7a). The scheme on Fig. 7b demonstrates the principle of rapid change of tubes direction in hydrocolloids and gels where the beginnings of transverse tubular structure becomes the dominant direction in the tubes alignment.

### Cytotoxicity study of hyaluronic acid/MAg NPs gel - potential application as anticancer drug

#### Cytotoxicity on 2D and 3D HeLa cell cultures

The cytotoxicity study was performed *in vitro* on cervical cancer cell line (HeLa) in order to reveal the potential in cancer treatment of MAg NPs and hyaluronic acid/MAg NPs gel. In our study, 2D cell culture model was used to provide basic information on cytotoxicity, penetration and accumulation of NPs in cells, whereas 3D cell cultures were used to obtain more accurate information about NPs behavior in living tissue^41,42^.

At the first stage of the study, an effect of different concentrations (10–1000 µg/ml) of MAg and USIO (used for comparison) NPs in aqueous dispersions after 24 and 48 h of exposition was studied on 2D cell culture. USIO NPs revealed low toxic effect (LD50 ∼1000 µg/ml) after 24 h, but increase of toxicity after 48 h of exposition (LD50 ∼500 µg/ml) (Supplementary Fig. S12a). In contrast to USIO NPs, MAg NPs significant toxic effect, ranging from concentration of 50 µg/ml (viability ∼30%) after 24 h and of 10 µg/ml (viability ≤30%) after 48 h, was revealed. As known, iron oxide NPs are biocompatible and do not reveal toxic effect even at significantly high concentrations^9,43^. Also, our previous results showed that MAg NPs produced with *Ginger rhizome* extract revealed low cytotoxicity even at a concentration of 200 µg/ml (HeLa cells viability ∼90%)^9^. These results allowed us to conclude that (i) the toxic effect of USIO NPs after 48 h of exposition is due to *Amanita muscaria* compounds on the NPs surface and (ii) the toxic effect of MAg NPs may be attributed to both silver and *Amanita muscaria* compounds activities.

**Figure 8:**
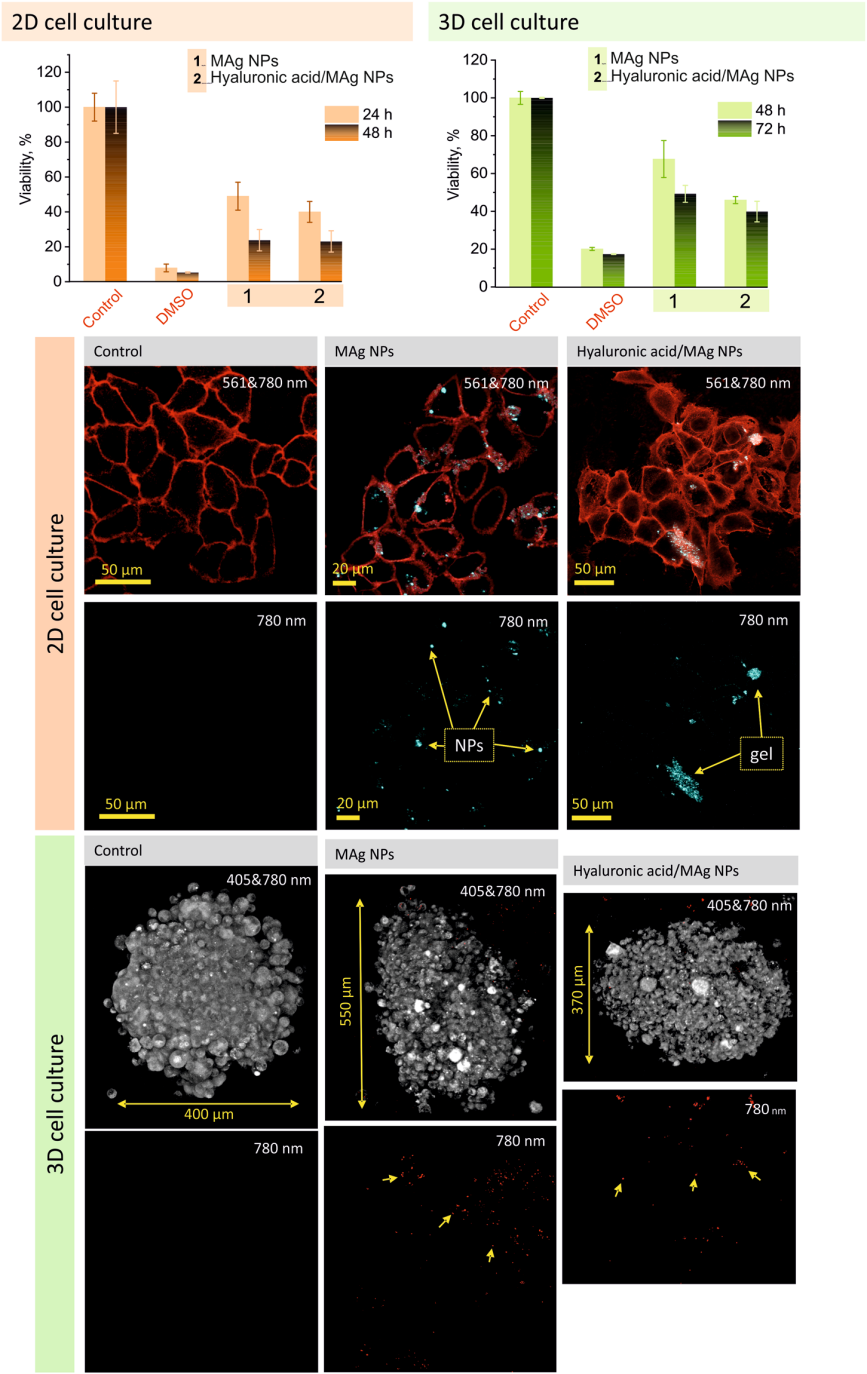
Cytotoxicity study on 2D and 3D HeLa cell cultures: viability after exposition with MAg NPs and hyaluronic acid/MAg NPs gel (MAg NPs - 25 µg/ml, hyaluronic acid - 5 mg/ml) (n = 3 for 2D cell culture, n = 9 for HeLa spheroids, ± SD); fluorescence images taken after 3 h of exposition (2D, excitation wavelengths 561 (cells, red) and 780 nm (MAg NPs, cyan)) and 24 h (3D, excitation wavelengths 405 nm (autofluorescence of cells, grey) and 780 nm (MAg NPs, red)). Representative images of three HeLa spheroids are shown.

On the next stage of cytotoxicity study, USIO and MAg NPs were studied at the same concentration range but dispersed in hyaluronic acid gel. The viability of cells after interaction with blank hyaluronic acid gel (5 mg/ml) was ∼90% that confirmed non-toxic properties of hyaluronic acid (see Supplementary Fig. S12a). Hyaluronic acid/USIO NPs gels revealed moderate toxic effect (LD50 ∼250 µg/ml) after 24 h, and low toxic effect after 48 h of exposition (viability ∼80% for all concentration range). Hyaluronic acid/MAg NPs gels revealed significant effect ranging from concentration of 50 µg/ml (viability ≤35%). One may notice that hyaluronic acid reduces toxicity of MAg NPs. As cytotoxic effect of MAg NPs was observed from concentration of 50 µg/ml for both aqueous and hyaluronic acid dispersions, lower concentration (25 µg/ml) was chosen for further tests. As seen, a concentration of 25 µg/ml of MAg NPs caused similar cytotoxic effect (48 h) in aqueous dispersion and in gel formulation (∼23% of viability). Fluorescence imaging of HeLa 2D cells (Figs 8 and S13b) performed after 3 h of exposition with MAg NPs aqueous dispersion and hyaluronic acid/MAg NPs gel showed that MAg NPs in aqueous dispersion were distributed between and inside cells; hyaluronic acid/MAg NPs gel was not completely solved (gel pieces were visible). This observation allowed us to conclude that (i) the MAg NPs could penetrate inside cells and (ii) slow gel dissolution may provide prolong therapeutic effect.

The next set of experiments was performed on 3D cell culture – HeLa spheroids. The results of the cellular spheroids viability showed that they were significantly more resistant to the cytotoxic action of MAg NPs and their gel formulation than 2D cell culture. Moreover, MAg NPs in gel formulation appeared to be more effective than MAg NPs in aqueous dispersion (48 h viability were ∼45 and ∼68%, respectively) (Fig. 8). Fluorescence imaging of spheroids after interaction with the samples (24 h) revealed that, in comparison with control where the cells are densely packed, the spheroids became friable and some holes are visible between cells (Fig. 8). Fluorescence images taken on different spheroid depth (slices of 3D scans) confirm the penetration of MAg NPs inside spheroids (Supplementary Fig. S13).

A discrepancy between the results obtained on 2D and 3D cell cultures may be related to the difference in cell architecture − 2D represents non-natural cell morphology where the cells are in the same phase of the cell cycle, whereas 3D culture better mimics biological tissue. Morphologically, the spheroids composed from cells with different phenotypes which are in the quiescence phase of the cell cycle, from necrotic cells in the center to dividing cells in the surface layers. Difference in 2D and 3D cytoarchitecture resulted in different gene expression that can fundamentally change cell behavior^41,42^. As known, data obtained using traditional 2D cell cultures *in vitro* poorly predict *in vivo* results^44–46^. 3D cell culture (also called organoids) is considered to be a bridge between traditional cell cultures and *in vivo* models^44,47^ and may reduce a gap existing between them. HeLa spheroids are characterized as culture that more accurate represents the cytoarchitecture of tumor tissue and exhibit characteristic features of its growth in the early stage *in vivo*. In connection with this, higher effectiveness of hyaluronic acid/MAg NPs gel comparing to MAg NPs in aqueous dispersion towards HeLa spheroids may be related to the difference in cell gene expression and increased need of hyaluronic acid^21^ that resulted in improved uptake of MAg NPs. To summarize, our results pointed to a high potential of hyaluronic acid/MAg NPs gel for local treatment of cancer. A formulation with hyaluronic acid gives advantages not only in manipulation, providing prolong local therapeutic activity, but also improves NPs transport inside cancer spheroids.

#### Two ways to improve therapeutic activity of anticancer gel

We suggested two possible ways that may improve the therapeutic effectiveness of hyaluronic acid/MAg NPs gel: (i) the introduction of additional component into gel and (ii) the use of laser irradiation in order to activate photoactive properties of silver NPs.

**Figure 9:**
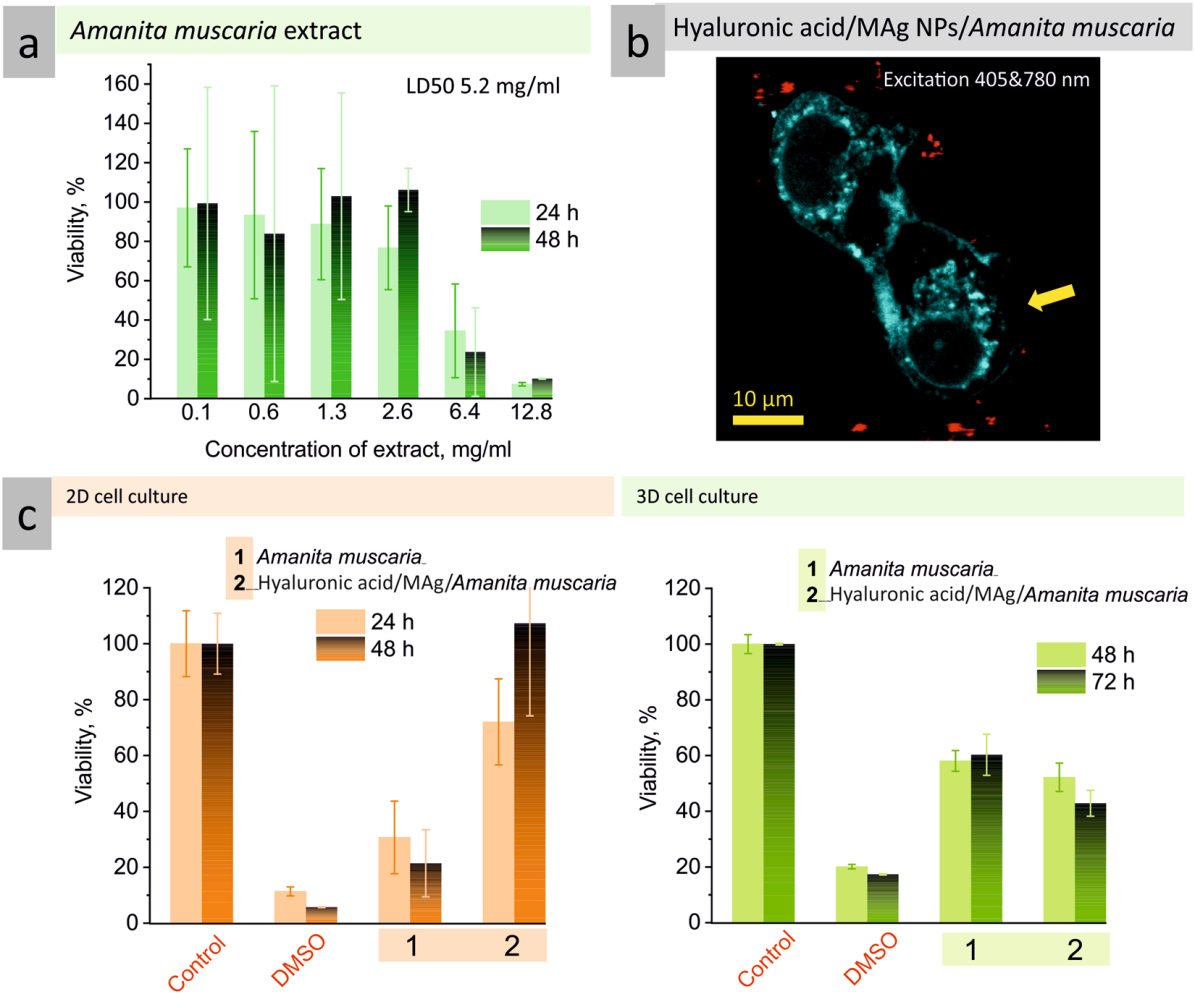
2D HeLa cells viability after exposition with *Amanita muscaria* extract (extract concentration was estimated by weighing of extract dry residue (dried at 50 °C) (**a**); fluorescence images of 2D HeLa cells after 24 h of exposition with hyaluronic acid/MAg/*Amanita muscaria* gel (excitation wavelengths 405 nm (autofluorescence of cells, cyan) and 780 nm (MAg NPs emission, red)), the arow points to the broken membrain of cell) (**b**); viability of 2D and 3D HeLa cell cultures after exposition with *Amanita muscaria* extract (7.5 mg/ml) and hyaluronic acid/MAg/*Amanita muscaria* gel (hyaluronic acid - 5 mg/ml, MAg NPs - 25 µg/ml, *Amanita muscaria* – 7.5 mg/ml) (**c**).

(i) Gel formulation of MAg NPs provides attractive opportunity to introduce additional component to the formulation; we supposed that addition of *Amanita muscaria* extract would improve therapeutic effect. To realize this idea, the toxicity of *Amanita muscaria* extract towards 2D HeLa cell culture was investigated first. It was found that cytotoxicity of *Amanita muscaria* extract was >100 times lower (LD50 ∼5.2 mg/ml) than that for MAg NPs (Fig. 9a). To check the influence of different concentration of *Amanita muscaria* extract, we added it to hyaluronic acid/MAg NPs gel where the concentration of MAg NPs was kept constant (25 µg/ml) (Supplementary Fig. S14a). The experiment was performed in comparison with hyaluronic acid/*Amanita muscaria* gel (without MAg NPs). The results were contrary to our expectations: addition of *Amanita muscaria* extract eliminate the toxicity of hyaluronic acid/MAg NPs gel; HeLa cells viability after 48 h of exposition was even higher than in negative control cells (≥100%). It is possible that for such a formulation higher concentration of NPs is needed. Therefore, in the next set of experiments, we tested hyaluronic acid/MAg NPs/*Amanita muscaria* gels which contained MAg NPs in a concentration range of 10–1000 µg/ml and constant amount of *Amanita muscaria* extract (7.5 mg/ml) (see supplementary Fig. S14a). Surprisingly, in the presence of *Amanita muscaria* extract, the gel exhibited toxicity only at MAg NPs concentration of 500 µg/ml which is 10 times higher than for MAg NPs without extract (HeLa cells viability ∼40 and <20% after 24 and 48 h, respectively). These confused results may also be connected with methodology of experiment - it is possible that duration of experiment was not sufficient to reveal long-term effect of such gel formulation. Fluorescence imaging of 2D HeLa cell culture after 24 h of exposition with hyaluronic acid/MAg NPs/*Amanita muscaria* gel revealed the cells with membrane perturbation (Fig. 9b), whereas for MAg NPs aqueous dispersion and hyaluronic acid/MAg NPs gel the only dead cells were observed (Supplementary Fig. 14b).

**Figure 10:**
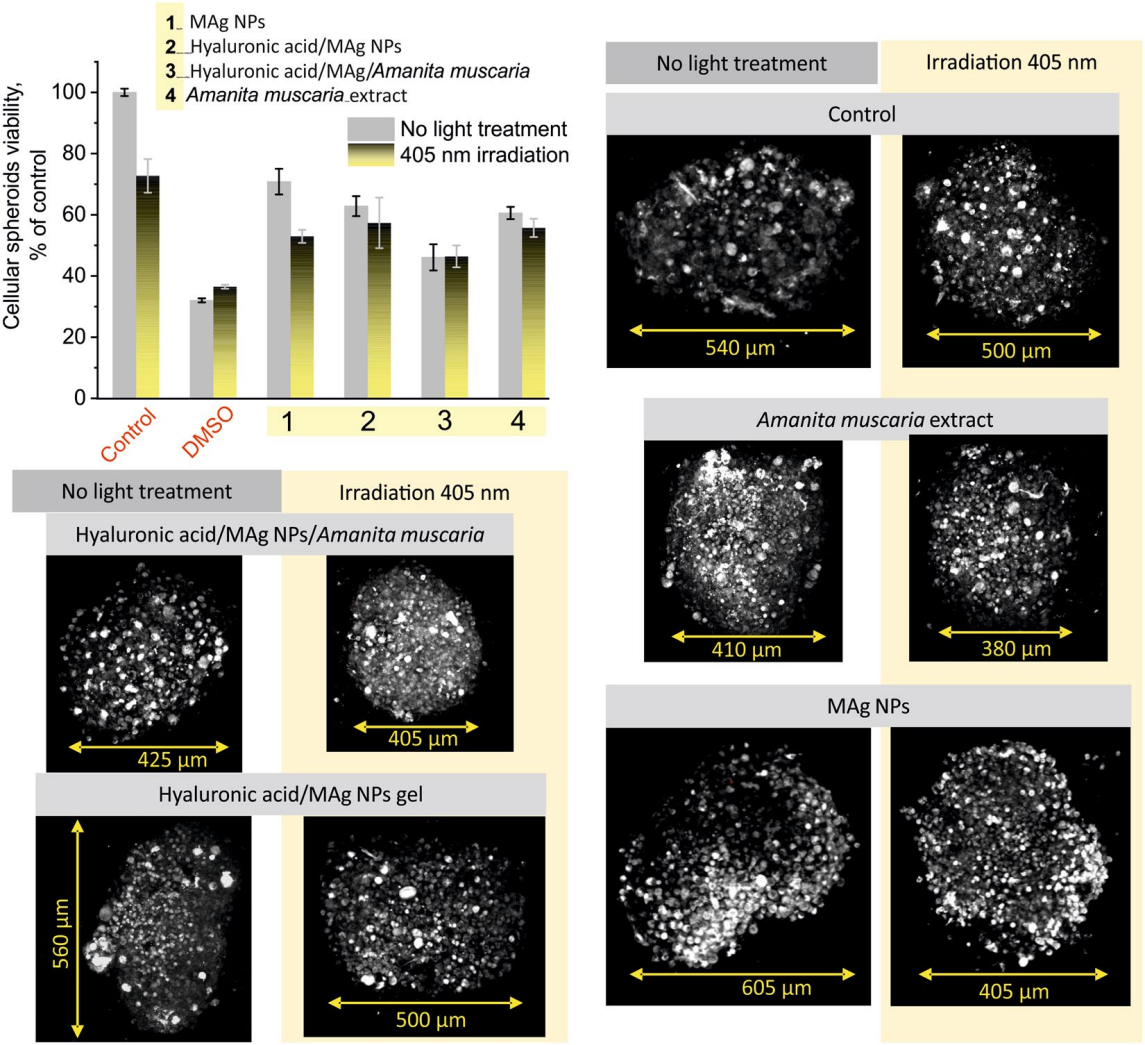
Influence of laser irradiation on cytotoxicity: viability and fluorescence 3D images of HeLa spheroids (n = 9, ± SD; representative images of 3 spheroids are shown), excitation wavelengths 405 nm (autofluorescence of cells, grey) and 780 nm (MAg NPs emission, red).

Cytotoxicity study performed on HeLa spheroids showed that 3D HeLa cell culture was twice more resistant (if compare to 2D cell culture) to cytotoxic properties of *Amanita muscaria* extract (48 h viability 57 and 21%, respectively) (Fig. 9c). And *vice versa*, for gel formulation with *Amanita muscaria* extract HeLa spheroids viability was decreased twice as much as for 2D cell culture (48 h viability 52 and 112%, respectively). As seen, the cell response was drastically different for 2D and 3D cell culture. The explanation of this fact was given above (different cytoarhitecture and gene expression). These results allowed us to conclude that (i) 2D cell culture is reasonable to use in a preliminary study only and (ii) hyaluronic acid in gel formulation improves uptake of active substances. We also supposed that such results may be connected with immunostimulation properties of *Amanita muscaria*^15^ and therapeutic effect of hyaluronic acid/MAg NPs/*Amanita muscaria* gel may be significantly better in time. Investigation of immune response caused by the *Amanita muscaria* compounds as well as increasing the duration of the experiment (which is possible for 3D cell culture) may provide an answer for this question and is worth to be performed in future study.

(ii) Laser irradiation with wavelengths 405 nm was applied in order to activate photoactive properties of silver NPs in gels providing better therapeutic results. This experiment was performed on 3D HeLa cell culture. Hyaluronic acid/MAg NPs (hyaluronic acid – 5 mg/ml, MAg NPs – 25 µg/ml), hyaluronic acid/MAg NPs/*Amanita muscaria* (hyaluronic acid – 5 mg/ml, MAg NPs – 25 µg/ml, *Amanita muscaria* – 7.5 mg/ml) gels, MAg NPs aqueous dispersion (25 µg/ml) and *Amanita muscaria* extract (7.5 mg/ml) were used. The results showed that light irradiation decreases the viability of cells in spheroids when applied to MAg NPs (on 18%), hyaluronic acid/MAg NPs gel (on 5%) and *Amanita muscaria* extract (on 5%) (Fig. 10). No effect was observed for hyaluronic acid/MAg NPs/*Amanita muscaria* gel. Fluorescence imaging performed after 24 h of initial irradiation revealed slight decrease in spheroids size in comparison with non-irradiated samples (see Fig. 10). These results showed that laser irradiation of hyaluronic acid/MAg NPs gel could be a promising technique to improve local therapeutic action (e.g., for treatment of acute or drug resistant cancer types).

Taking into account the possible interaction of hyaluronic acid/MAg NPs gel with normal cells, the cytotoxic effect was studied also on human fibroblast cell line MSU-1.1. Quite expectedly, the results showed high cytotoxicity for normal 2D cell culture (viability was ∼12%) (Supplementary Fig. S15). Such a high cytotoxicity towards normal cell lines is typical for anticancer drug, e.g., doxorubicin inhibits fibroblasts migration and proliferation even at concentration 10^−6^ M (0.5 µg/ml), whereas doses at 5·10^−5^ M and higher showed cellular toxicity and effectively inhibits wound repair^47–49^. Though the study on 3D fibroblast culture may give different result, the only local application of hyaluronic acid/MAg NPs gel may be considered.

## Conclusion

On the basis of hyaluronic acid, we developed gel formulation with iron oxide (∼2 nm) and silver (5–25 nm) NPs for local anticancer therapy. *Amanita muscaria* extract was used for the synthesis of MAg NPs as capping agent. *Amanita muscaria* compounds formed surface layer of NPs containing glucans and muscimol, which significantly impacted anticancer properties.

Cytotoxicity studies performed on 2D and 3D HeLa cell cultures pointed to a high potential of hyaluronic acid/MAg NPs gel for local treatment of cancer. Hyaluronic acid used as gelling agent in such formulation was found to increase an effectiveness of active components (MAg NPs, *Amanita muscaria* extract) probably improving their transport inside HeLa spheroids. Moreover, gel formulation gives advantages in manipulation and provides prolong local therapeutic activity. In our study, cell response was drastically different for 2D and 3D cell culture that was related to their different cytoarhitecture and gene expression. Thus, the results of the cellular spheroids viability showed that they were significantly more resistant to the cytotoxic action of MAg NPs and their gel formulation than 2D cell culture.

We also proposed two possible ways to improve anticancer activity of hyaluronic acid/MAg NPs gel that may be promising for local treatment of acute or drug resistant cancer types: first, the introduction of additional active component into the gel, and second, use of laser irradiation to activate the photoactive properties of silver NPs. In spite of the fact that our attempt to introduce additional active component (*Amanita muscaria* extract) was not highly efficient, this approach promises a lot of advantages and is worth to be developed. Introduction into the gel some of contrast agents, novel anticancer or pain reliever drugs looks to be very useful. Using of laser irradiation (405 nm) turned to be another effective instrument to improve local therapeutic action. Presence of iron in the gel composition would allow to monitor the treatment process using magnetic resonance imaging, which would be a topic of our future study.

Using cryo-SEM technique, we investigated MAg NPs hydrocolloids and gels microstructures, and found a high level of similarity and ordering between them. Our findings break the stereotype of gel structures as chaotic networks of fibers, but in fact their microstructure was well-ordered and consisted of perforated tubes with parallel alignment. The microstructure presented important similarities to skeletal muscular tissue (*Sus scrofa domesticus*) and therefore, hyaluronic acid/MAg NPs gel may also impact on the biocompatibility of *in vivo* experiments providing a scaffold for reconstruction of damaged tissue.

## Methods

### Materials

The analytical grade reagents of iron (III) chloride (FeCl_3_), iron (II) chloride tetrahydrate (FeCl_2_·4H_2_O), silver nitrate (AgNO_3_), ammonium hydroxide (NH_4_OH), ethanol, hyaluronic acid sodium salt from *Streptococcus equi*, bovine serum albumin (BSA) and phosphate buffered saline (PBS) (tablets) were purchased from Sigma-Aldrich and used as received. BSA solution (1 wt%) in PBS was used throughout the experiments. Reagents for biological tests including Dulbecco’s modified Eagle’s medium (DMEM), Hanks Balanced Salt solution, fetal bovine serum (FBS), Trypsine-EDTA (0.25%), penicillin-streptomycin, and agarose were purchased from Sigma-Aldrich. WST-1 Cell Proliferation Assay Kit was purchased from Clontech. Tetramethylrhodamine conjugate of Concanavalin A **(**TRITC-Con A) staining dye was obtained from Thermo Fisher Scientific. Formaldehyde 16% methanol-free was purchased from Polyscinces. Ultrapure water (resistivity >17 MΩcm) from a GZY-P10 water system was used throughout the experiments. For preparation of *Amanita muscaria* extract, seven fruit bodies were collected in Morasko forest (Poznan, Poland) in October 2017. Fresh domestic pig muscle tissue (Poland) was bought in local supermarket in Poznan, Poland.

### MAg synthesis

The synthesis of MAg NPs were performed via co-precipitation technique described previously^8^ with some modifications. To prepare *Amanita muscaria* extract, seven fresh *Amanita muscaria* mushrooms (500 ± 20 g) were washed and cut into small pieces. Chopped *Amanita muscaria* mushrooms were kept in a water-ethanol solution (500 ml, 1:1 ratio) for 14 days (room temperature, dark place). Then, supernatant was vacuum filtered (Whatman filter paper) and stored in refrigerator (4 °C). The concentration of dry residue of *Amanita muscaria* extract (dried at 50 °C) was 16.2 mg/ml. After evaporation in vacuum evaporator Hei-Vap Advantage (Heidolph) the concentration was 72.0 mg/ml.

In typical synthesis, 198 mg of FeCl_2_·4H_2_0 and 324 mg of FeCl_3_ were solved in water (25 ml) and mixed with *Amanita muscaria* extract (16.2 mg/ml, 25 ml). AgNO_3_ (105 mg) was solved in water (30 ml) and added dropwise to the iron salts solution. Then, mixture of NaOH (5 M, 15 ml) and *Amanita muscaria* extract (16.2 mg/ml, 15 ml) was added dropwise under rigorous stirring; the reaction mixture immediately turned black. The reaction mixture was kept under stirring at room temperature (22 °C, 5 h, 1300 rpm). Then, the deposit was washed with water (following centrifugation, 24000 rpm, 40 min). The synthesis of solely ultrasmall iron oxide (USIO) NPs were performed using the above mentioned procedure but without the addition of silver nitrate.

MAg NPs were found to form stable water dispersions. At high NPs concentration (≥37 mg/ml), the hydrocolloid turned into thixotropic gel in time. Throughout the article, the samples were investigated in different states: as a powder (for XRD, SEM EDS, FTIR), as a aqueous dispersion (~1 mg/ml, optical density (OD)≤1) (for UV-Vis, Zeta-sizer, DLS, etc.), and as a hydrocolloid (37 ± 1 mg/ml) (for fluorescence emittance, cryo-SEM, etc.).

For preparation of hyaluronic acid/MAg NPs gels, hyaluronic acid was solved in sterile water first (5 mg/ml), and then calculated amount of MAg NPs hydrocolloid was added and mixed.

### Characterization techniques

#### Physicochemical techniques

X-ray diffraction (XRD) studies were conducted on an Empyrean diffractometer (PANalytical), using Cu Kα radiation (1.54 Å), a reflection-transmission spinner (sample stage) and PIXcel 3D detector, operating in the Bragg–Brentano geometry. The °2Theta scans were recorded at angles ranging from 10 to 95° with a step size of 0.007° and continuous scan mode.

Transmission Electron Microscopy (TEM) measurements and elemental analysis were performed using a JEM-ARM-200F High Resolution Transmission Electron Microscope (accelerating voltage of 200 kV) equipped with dispersive X-ray spectrometer. FFT and FFT filtered images of size and shape of USIO NPs were done using DigitalMicrograph.

The microstructure morphology of USIO and MAg NPs hydrocolloids was examined by cryogenic scanning electron microscopy (cryo-SEM) method (accelerating voltage 5 kV). The samples were cryo-fixed by plunging them into sub-cooled nitrogen (nitrogen slush) close to the freezing point of nitrogen at −210 °C. The optimal sublimation time for the MAg NPs hydrocolloids was found to be 40 min, including 15 min for the sublimation of the fractured part of the sample. For cryo-SEM measurements of domestic pig muscular tissue, optimal sublimation time of fractured part was 45–60 min. Elemental analysis of the powdered samples, as well as cryo-SEM elemental mapping, was performed using the energy dispersive microanalysis (EDS) mode of an X-ray equipped SEM applying voltage 15 kV (n = 4, mean ± SD). The studies were carried out using Scanning Electron Microscope JEOL 7001 F.

Ultraviolet–visible spectroscopy (UV-Vis) measurements were performed using a Lambda 950 spectrophotometer (PerkinElmer). Dynamic light scattering (DLS) and zeta-potential measurements of nanoparticles were performed on the Litesizer 500 particle Analyser (Anton Paar). The excitation spectra, emission spectra of the samples were obtained by FluoroSENS Spectrophotometer (GildenPhotonics).

The kinetic of gel dissolution was studied by monitoring of UV-Vis spectra of samples (100 µl of gel in 3 ml of solvent) at different times intervals from 2 to 330 min. Maximum intensities of obtained UV-spectra were used for building of kinetics curves.

The rheological measurements were performed on rheometer Malvern Kinexus pro + at room temperature (25 °C).

The Fourier transform infrared (FTIR) transmittance spectra were obtained using a Tensor 27 (Bruker Optics) spectrometer equipped with a global source and MCT detector. Samples were prepared using KBr as a matrix material, and mixed in proportions of 1 mg of sample to 200 mg KBr. A dried residue of *Amanita muscaria* extract (drying temperature 50 °C) was used for pellet fabrication.

Thermal stability of the samples was measured by thermogravimetric analysis (TGA) using TGA Q50 (TA Instruments). Experiment was performed from 25 °C up to 600 °C with a heating rate of 10 °C/min under nitrogen flow (60 ml/min). Samples were placed in platinum crucible.

Fluorescence measurements were performed by means of a laser scanning microscopy system LSM 780 (Zeiss) equipped with a femtosecond tunable infrared laser for two-photon excitation.

#### Cell culture

Human cervical cancer cell line (HeLa) was obtained from American Type Culture Collection (ATCC). Human fibroblast cell line MSU-1.1 was obtained from Prof. C. Kieda (CBM, CNRS, Orléans, France). Cells were cultured in a complete medium (Dulbecco’s Modified Eagle’s Medium (DMEM) supplemented with 10% fetal bovine serum (FBS), 100 units/ml penicillin, 100 μg/ml streptomycin and grown at 37 °C in humidified atmosphere containing 5% CO_2_.

The HeLa cellular spheroids were constructed using InSphero GravityTRAP Ultra-Low Attachment (ULA) microplates (PerkinElmer). Briefly, HeLa cells monolayer were cultivated as described above. When the cells reached about 70% confluence, they were harvested by trypsinization, resuspended in fresh medium and counted (TC20 automated cell counter, BioRad). Then 500 cells per well were seeded onto 96-well Gravity Trap ULA plates, centrifuged briefly and incubated (37 °C, 3 d) before experiments.

#### Cytotoxicity and photo-cytotoxicity assays

Cervical cancer cells (HeLa), HeLa cellular spheroids and normal fibroblasts (MSU-1.1) were used for *in vitro* cellular toxicity and/or photo-cytotoxicity study. Cells (6 × 10^3^ cells/ml) were seeded onto 96-well plates and incubated overnight (37 °C, 5% CO_2_). The medium in the wells was then replaced with fresh medium containing studied samples and incubated for 24, 48 or 72 h. The medium without NPs was used as a negative control.

The photo-cytotoxicity on spheroids was performed according to Flak *et al*.^50^. Here, the cellular spheroids after 24-hours incubation with NPs were washed with PBS and irradiated using near-visible light exposure (405 nm, power density of 2.30 mW/cm^2^, 40 min). After irradiation the PBS was exchanged back to DMEM and spheroids were incubated for next 24 h prior to viability assay.

The effect of the NPs on cell proliferation and viability was determined by WST-1 assay according to manufacturer’s instructions. Briefly, WST-1 solution (10 μl) was added to each well and the plates were further incubated. After 2 h, the absorbance was measured with a microplate reader (Anthos Zenyth 340rt) at 450 nm, and 650 nm as reference. The relative cell viability (based on cells metabolic activity) related to the negative control was calculated by test sample/negative control × 100%. Data are reported as the average ± standard deviation (SD) of wells performed in quadruplicate. In case of the 3D culture, the three spheroids per well in triplicates were used.

#### Confocal fluorescent imaging

HeLa cells were used for *in vitro* 2D visualization, while HeLa cellular spheroids for *ex vivo* 3D tumor visualization. Briefly, cells were placed on a chambered labtek dish (1 × 10^4^ cells/well), grown overnight, and then incubated with the samples for 24 h. The cells were then rinsed with PBS, fixed in 3.7% formaldehyde and stained with cell membrane glycoproteins TRITC-Con A (Thermo Fisher Scientific) dye. Next, cells were analyzed using a confocal laser scanning microscope (Zeiss LSM 780 NLO) in normal mode and 3D sectioning at excitation wavelengths 405 (cell autofluorescence), 633 (TRITC-Con A dye), and 780 (MAg NPs) nm.

## Electronic supplementary material

Supplemental data
