# Supplementary material for: Gel with silver and ultrasmall iron oxide nanoparticles produced with Amanita muscaria extract: physicochemical characterization, microstructure analysis and anticancer properties
Source: Sci Rep. 2018 Sep 5;8:13260. doi: 10.1038/s41598-018-31686-x (PMC6125601; doi:10.1038/s41598-018-31686-x)
Supplement: Supplementary file 1 — Supplemental data [file 41598_2018_31686_MOESM1_ESM.pdf]

Gel with silver and ultrasmall iron oxide nanoparticles produced with *Amanita muscaria* extract: physicochemical characterization, microstructure analysis and anticancer properties

Olena Ivashchenko[a]*, Łucja Przysiecka[a], Barbara Peplinska[a], Marcin Jarek[a], Emerson Coy[a], and Stefan Jurga[a]

SUPPLEMENTAL DATA

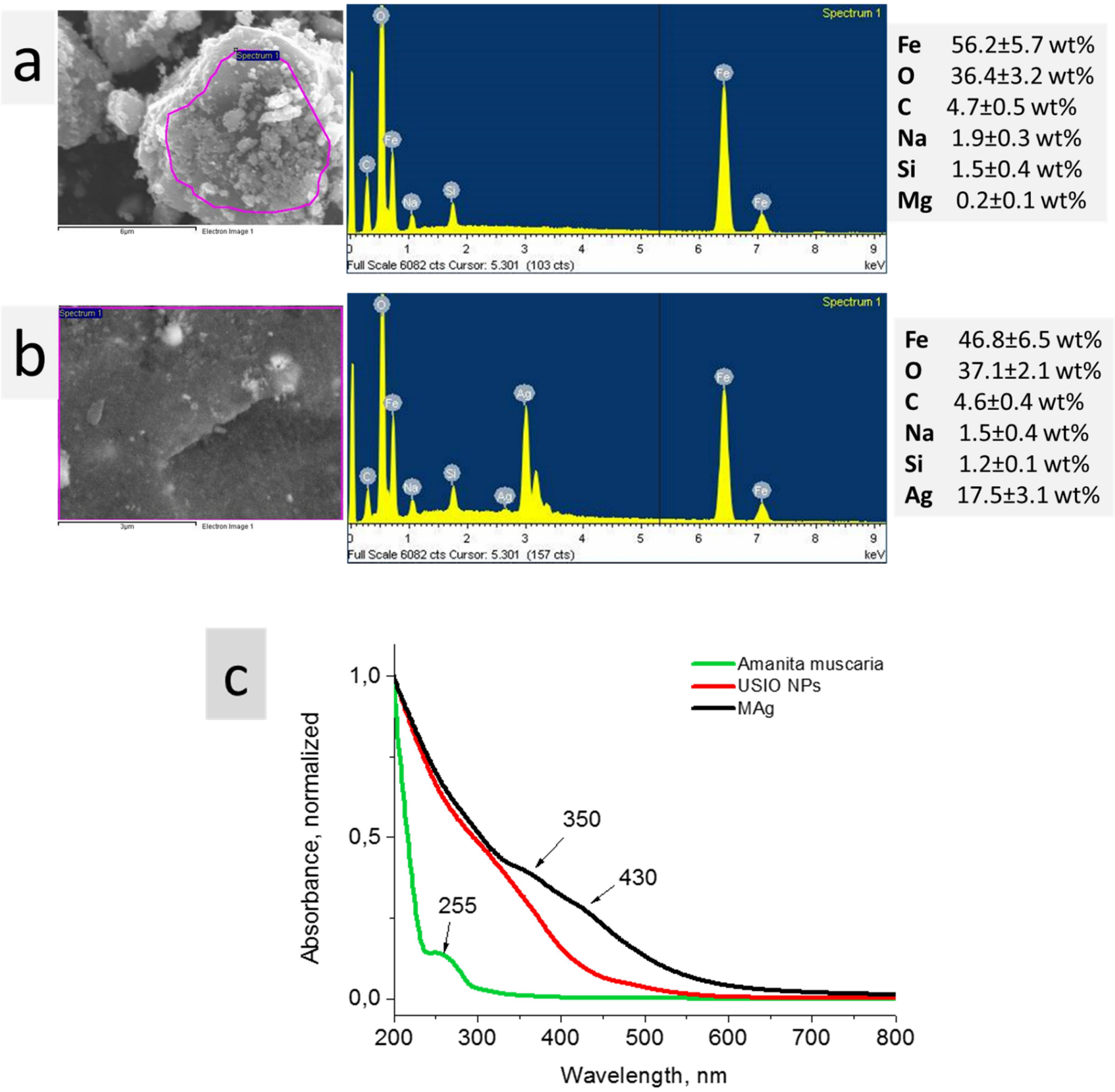

Fig. S1. Elemental composition of USIO (a) and MAg (b) NPs according to SEM EDS measurements; UV-Vis spectra of USIO, MAg NPs and *Amanita muscaria* (c).

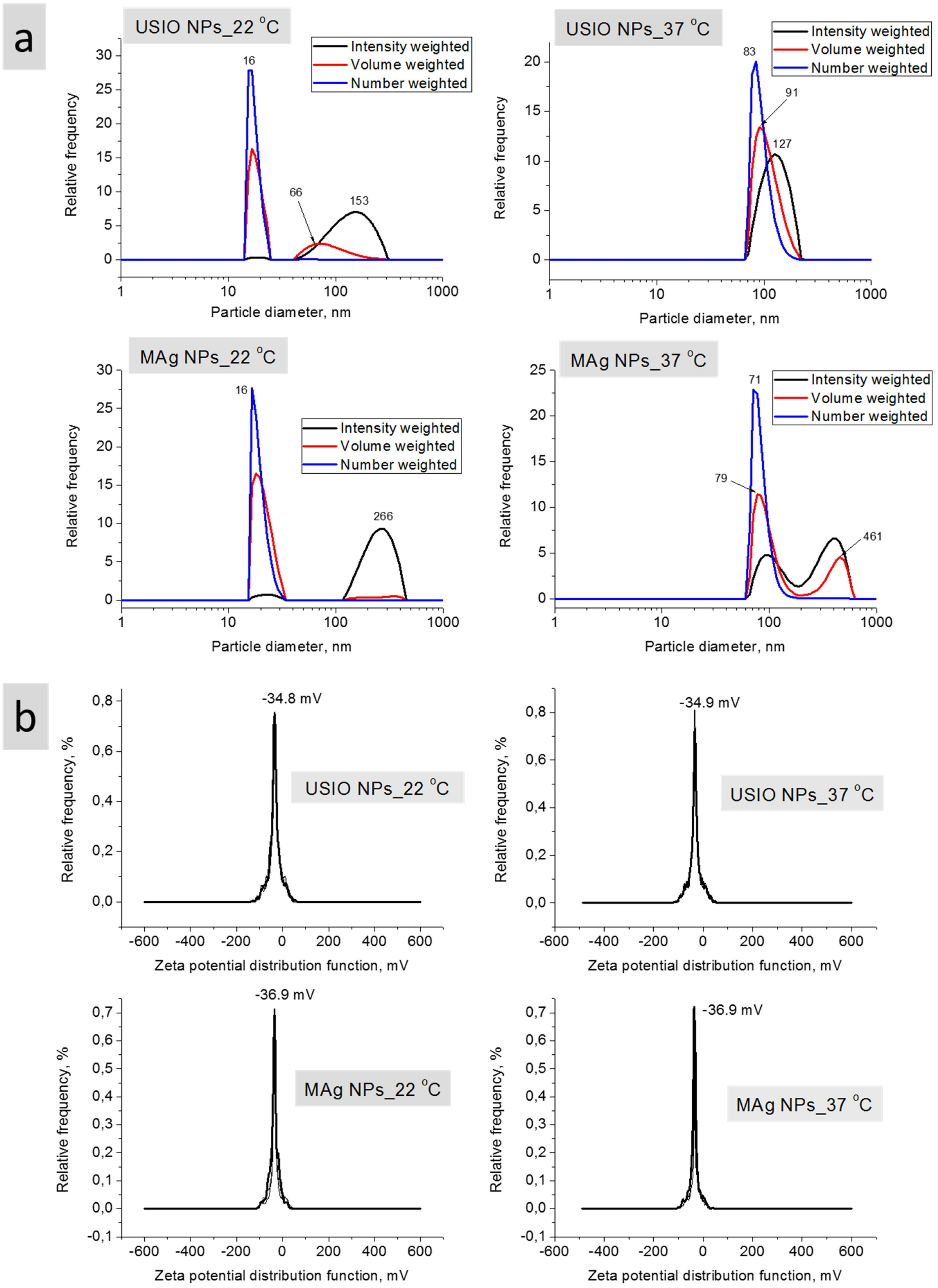

Fig. S2. DLS (a) and zeta-potential (b) measurements of USIO and MAg NPs in aqueous solutions at 22 and 37 °C.

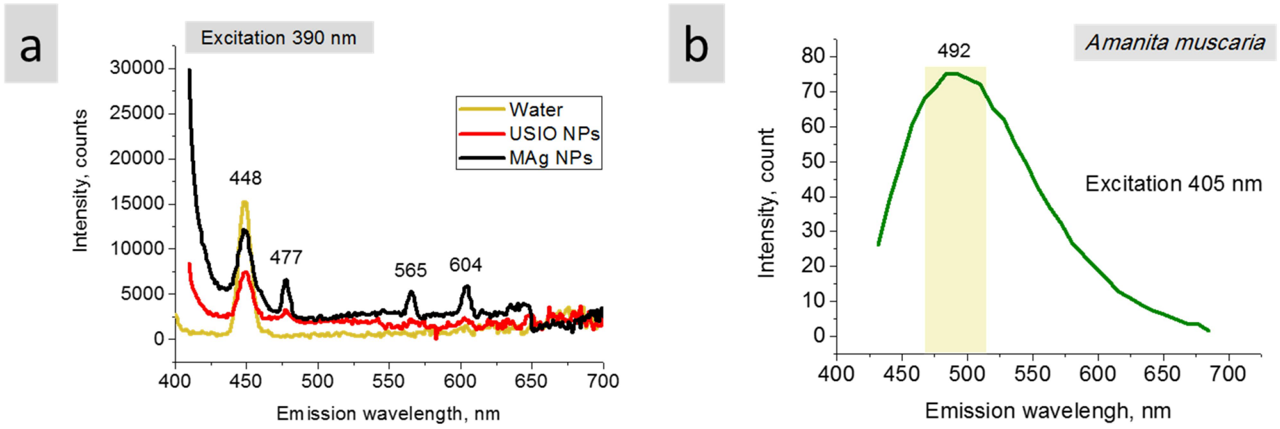

Fig. S3. Fluorescence emission spectra of USIO and MAg NPs aqueous diluted dispersions (OD≤1) measured by means of FluoroSENS Spectrophotometer (Gilden Photonics) (a) and *Amanita muscaria* extract measured by means of a laser scanning microscopy system LSM 780 (Zeiss, Germany) (b).

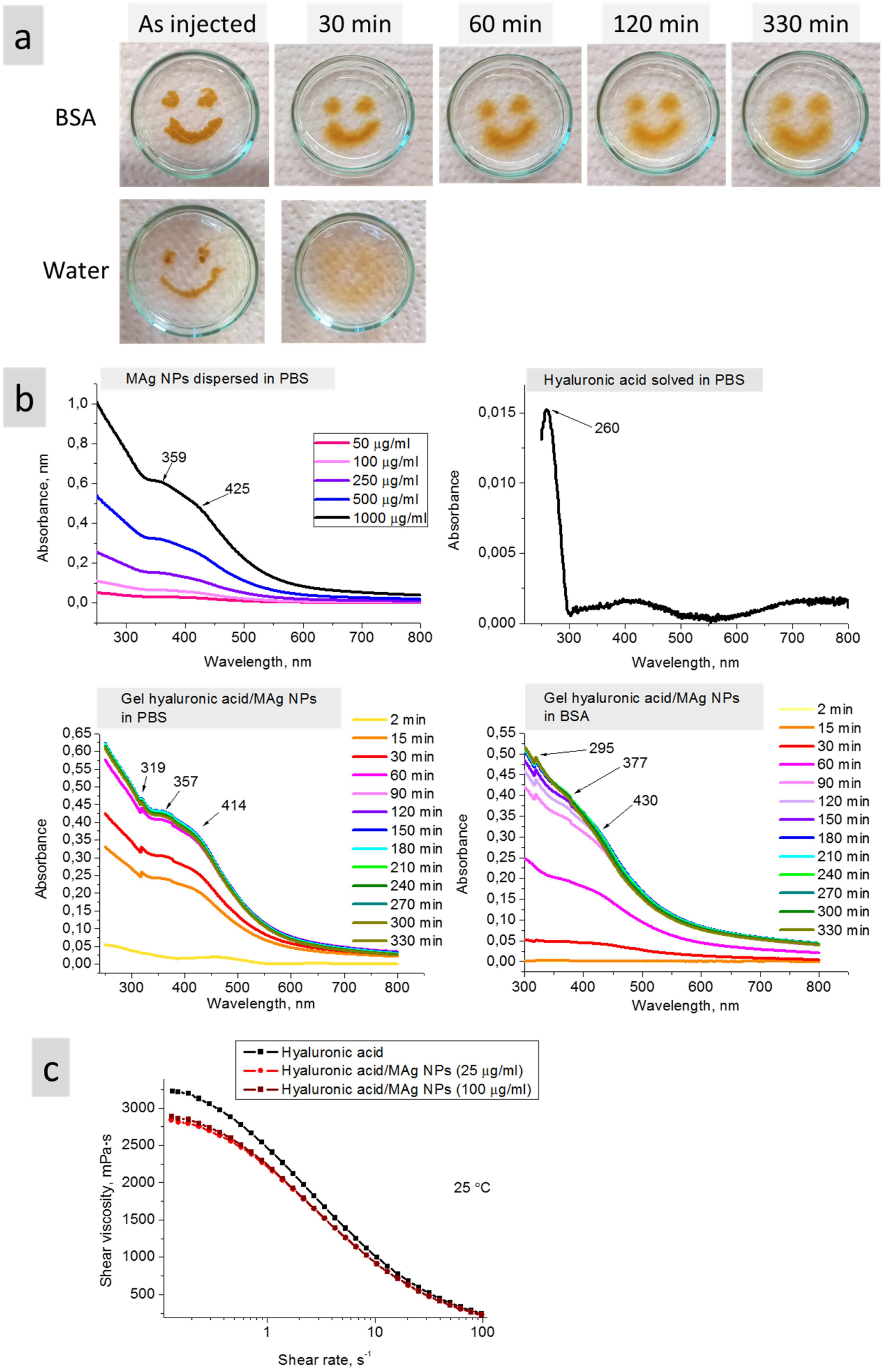

Fig. S4. Photos of hyaluronic acid/MAg NPs gel (MAg NPs concentration in gel was 1 mg/ml) injected to BSA solution at different time intervals after injection that demonstrate the gel solubility (a); source data for kinetic of gel dissolution calculation: UV-Vis spectra of MAg NPs dispersed in PBS, hyaluronic acid solved in PBS and hyaluronic acid/MAg NPs gel injected to PBS and BSA measured at different time intervals after injection (b); rheological measurements of hyaluronic acid and hyaluronic acid/MAg NPs gels (MAg NPs concentration 25 and 100 µg/ml) (c).

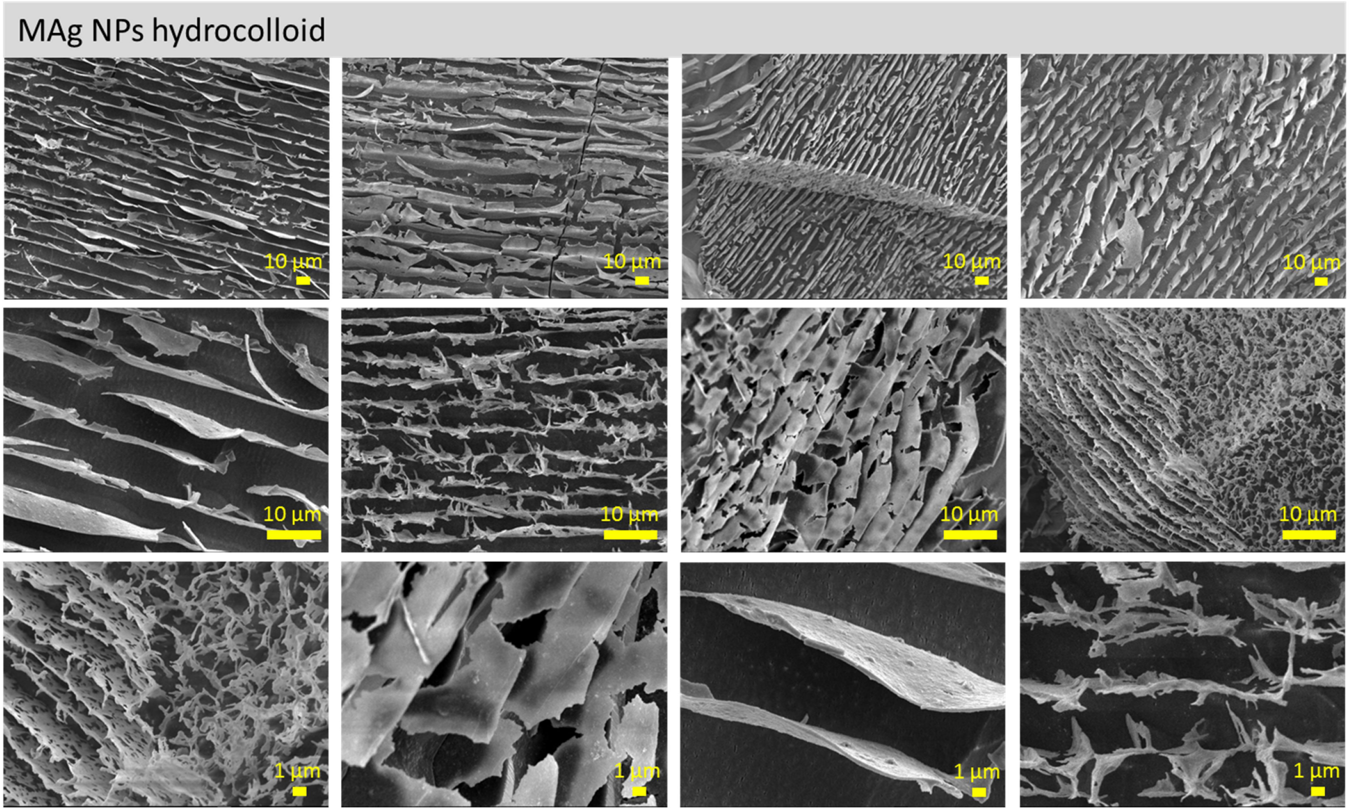

Fig. S5. Cryo-SEM images of MAg NPs hydrocolloid (37.1±1.1 mg/ml).

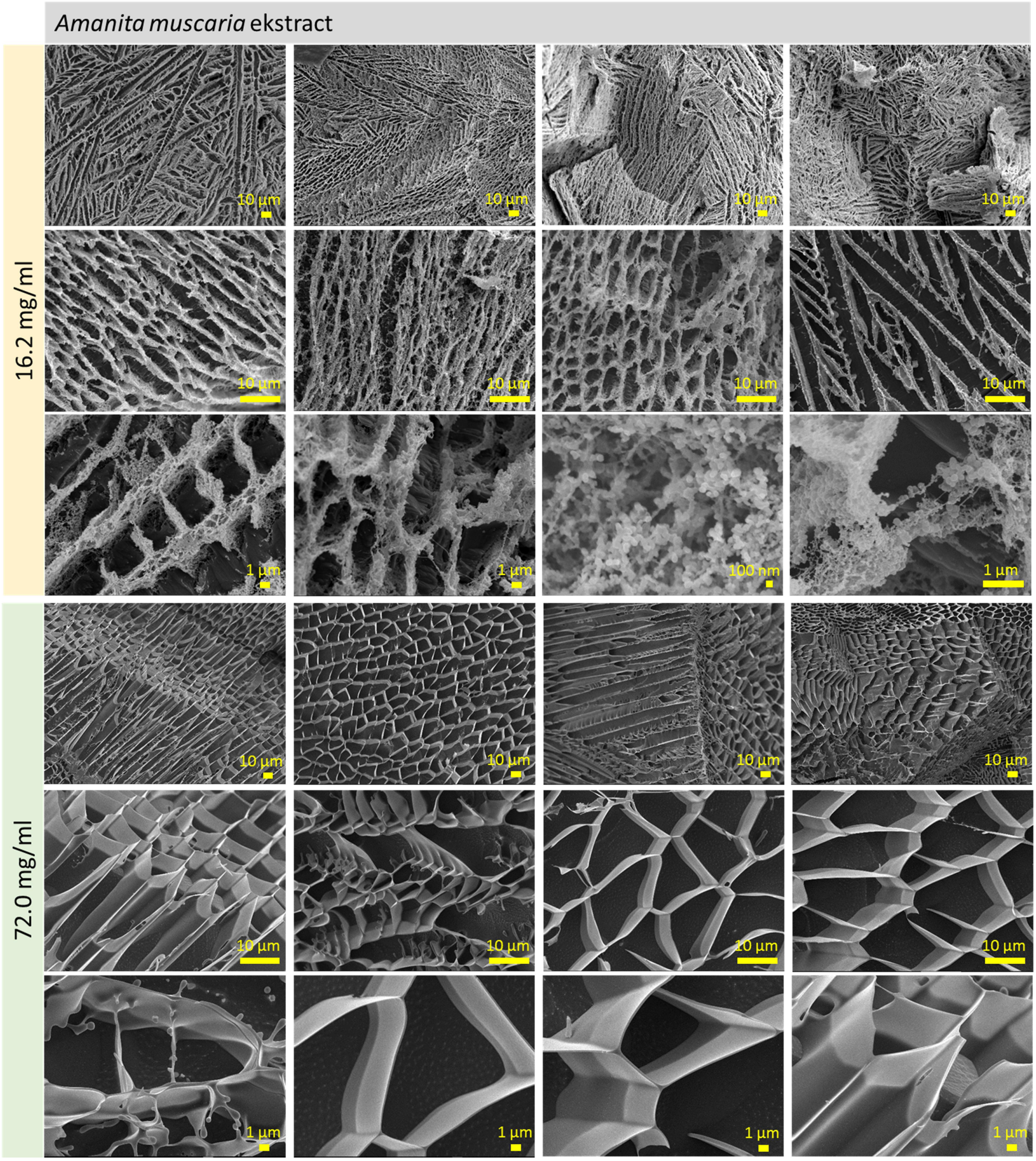

Fig. S6. Cryo-SEM images of *Amanita muscaria* extract with concentration 16.2 and 72.0 mg/ml.

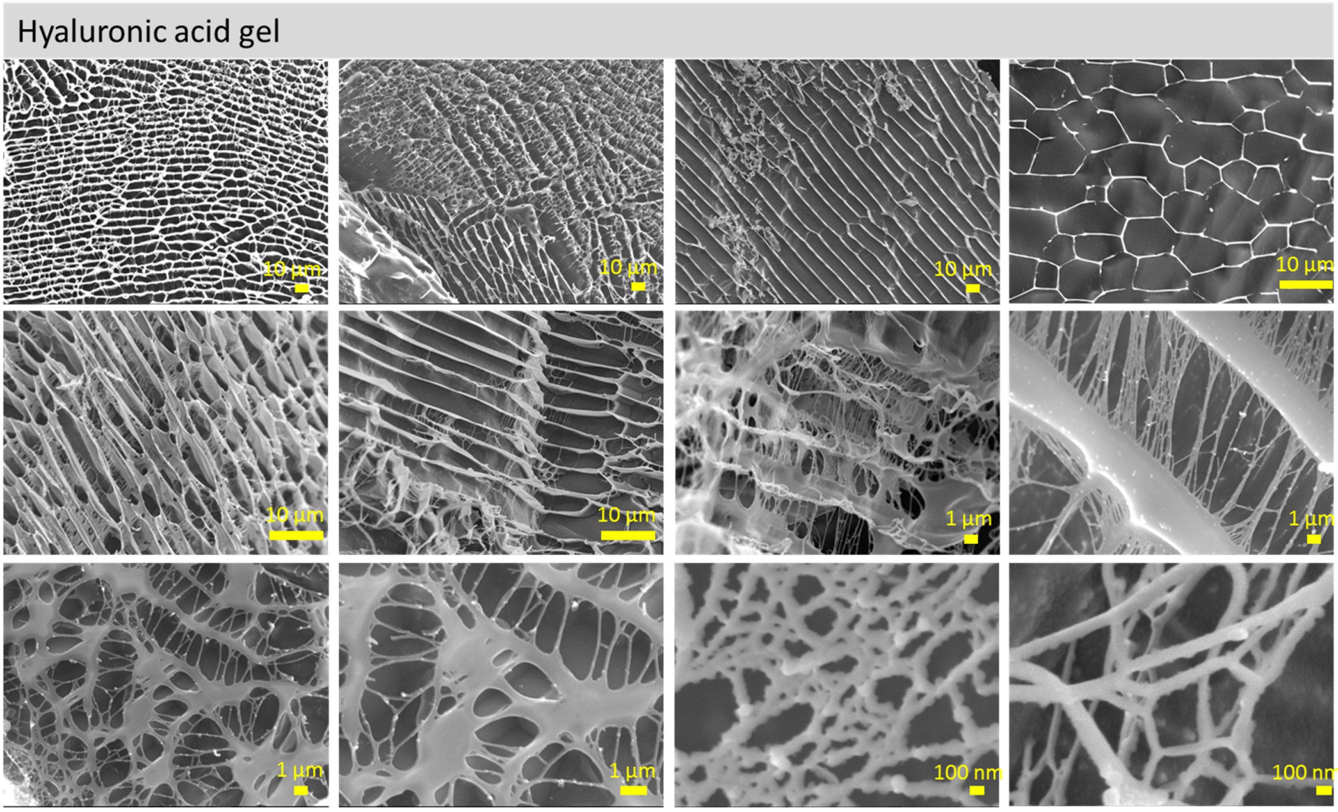

Fig. S7. Cryo-SEM images of hyaluronic acid hydrogel (5 mg/ml).

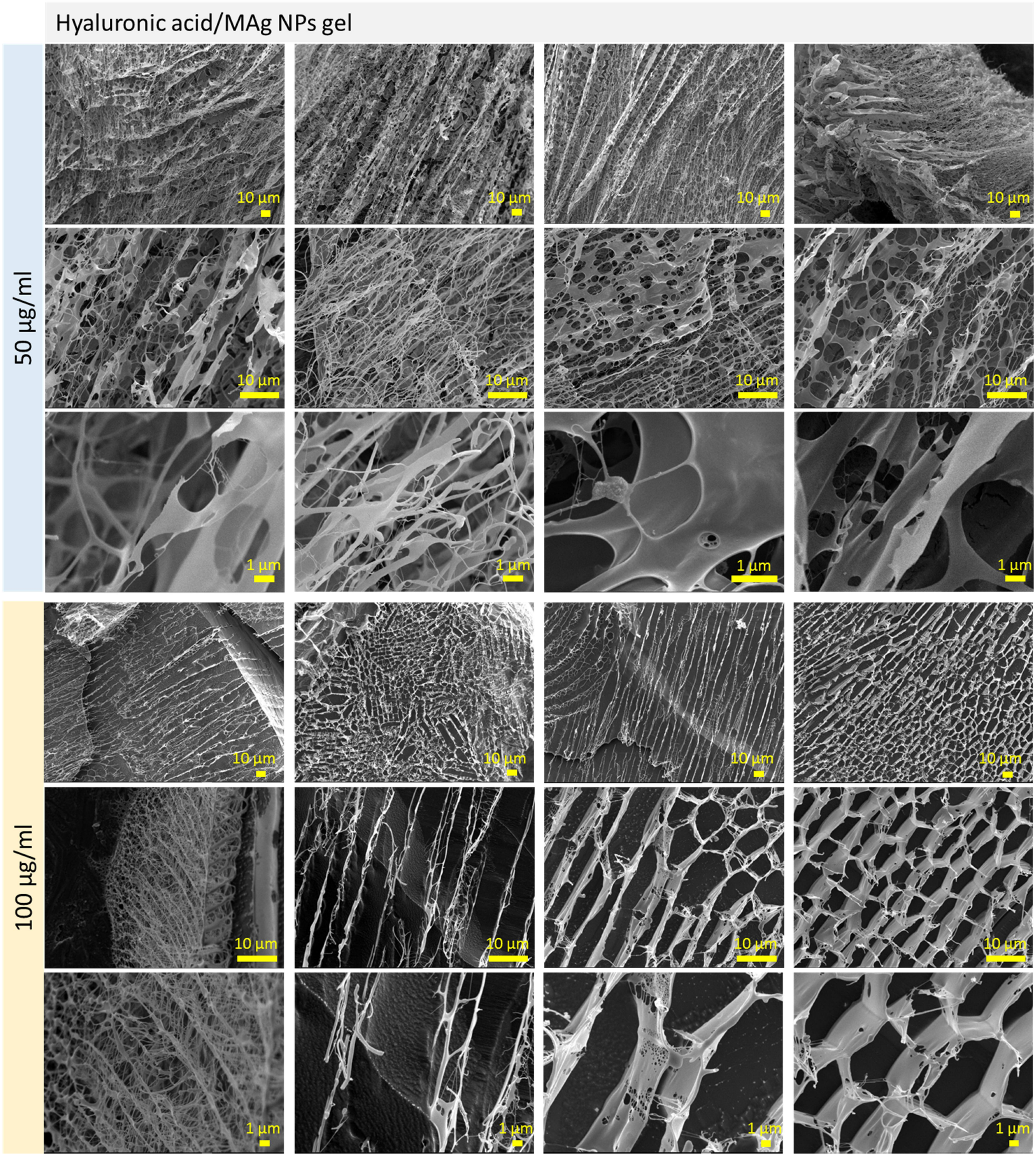

Fig. S8. Cryo-SEM images of hyaluronic acid/MAg NPs gel with nanoparticles concentration 50 and 100 µg/ml.

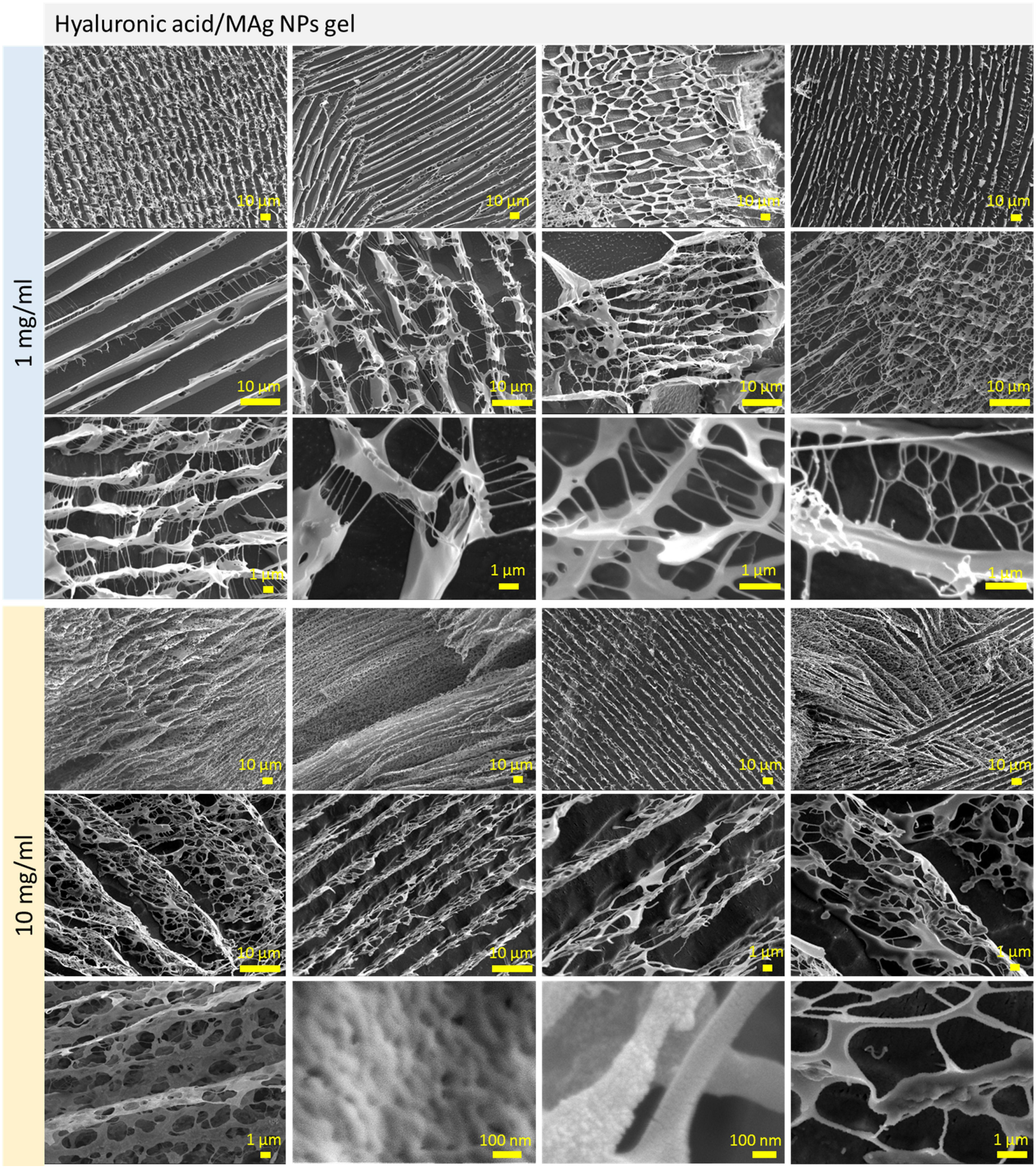

Fig. S9. Cryo-SEM images of hyaluronic acid/MAg NPs gel with nanoparticles concentration 1 and 10 mg/ml.

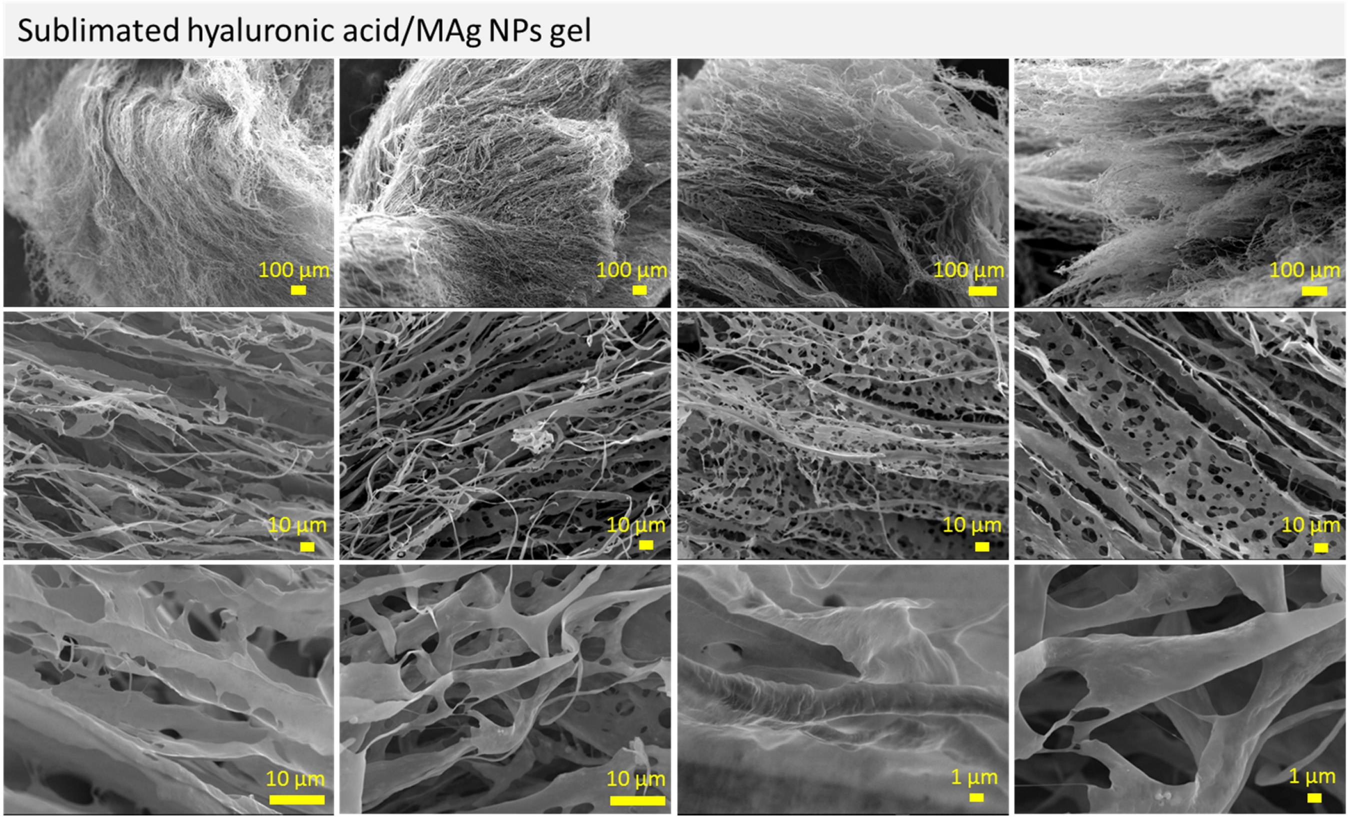

Fig. S10. SEM images of freeze-dried hyaluronic acid/MAg NPs gel (nanoparticles concentration 1 mg/ml, -85 °C, 4 days).

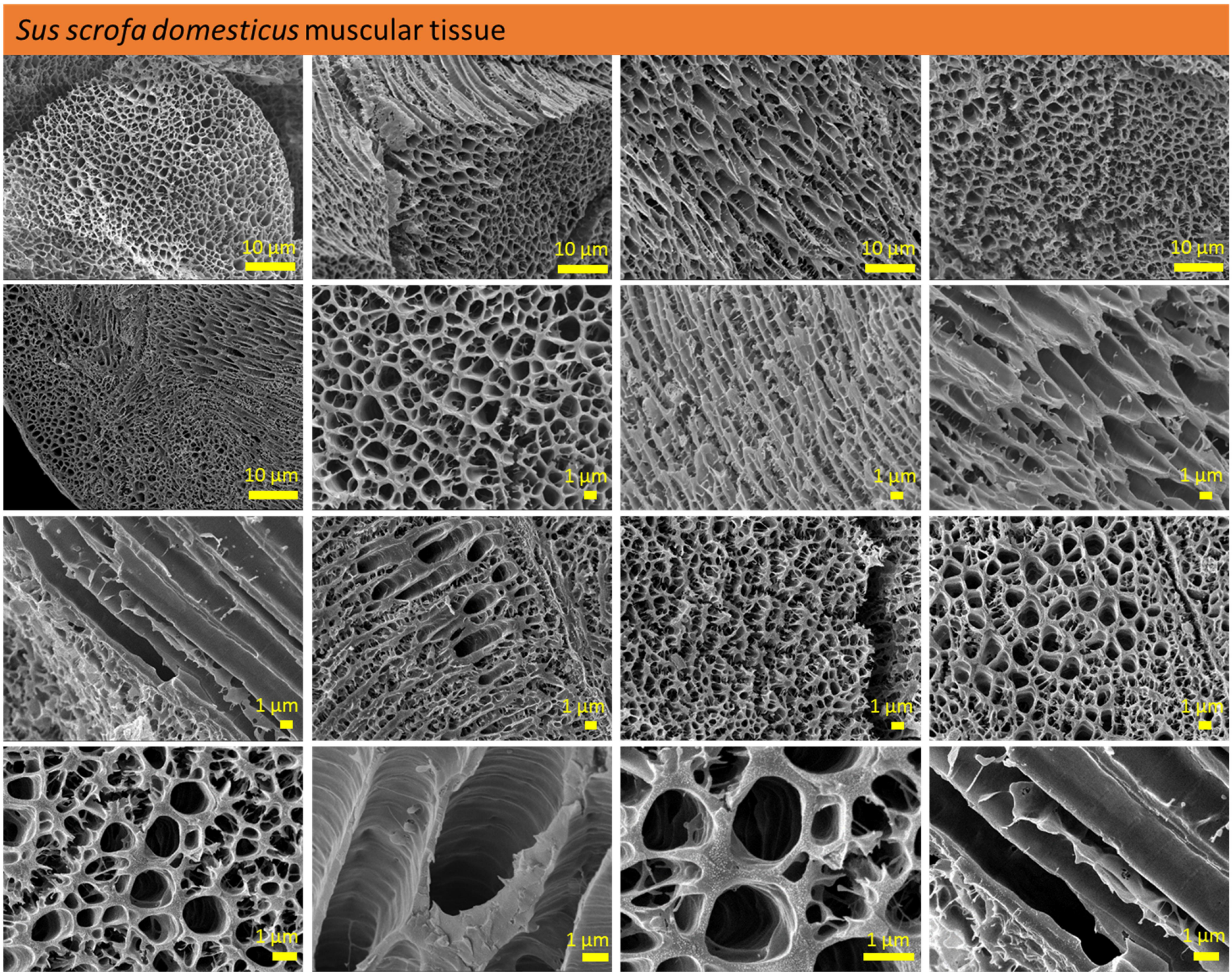

Fig. S11. Cryo-SEM images of *Sus scrofa domesticus* muscular tissue.

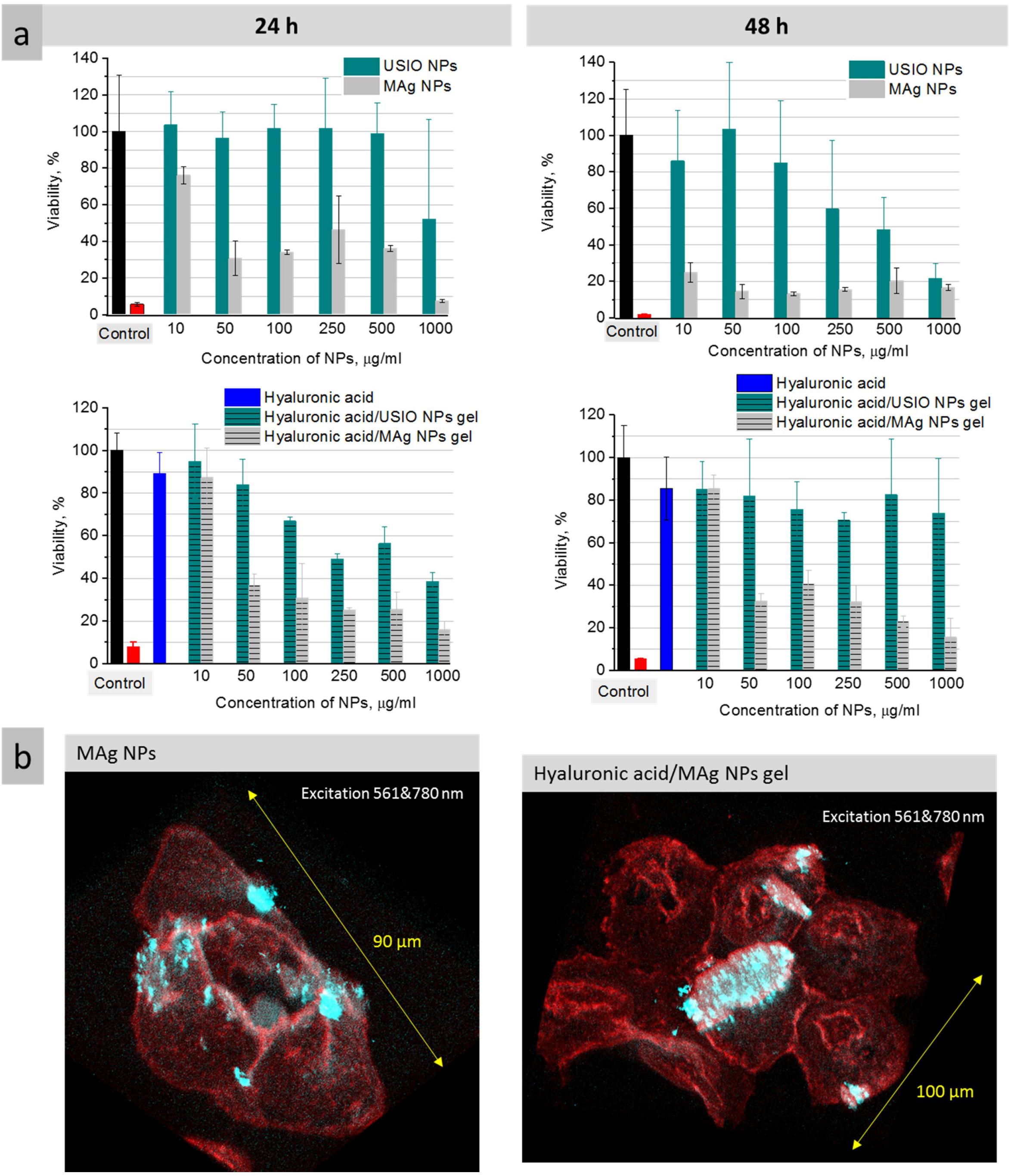

Fig. S12. Cytotoxicity study on 2D HeLa cell culture: viability after 24 and 48 h of exposition with hyaluronic acid (5 mg/ml) and USIO, MAg NPs, hyaluronic acid/USIO NPs and hyaluronic acid/MAg NPs gels with different concentrations of components (n=3, ±SD) (a); fluorescence images (3D scan) of HeLa 2D cell culture after 3 h of exposition with MAg NPs and hyaluronic acid/MAg NPs gel (MAg NPs concentration was 25 µg/ml) in comparison with control cells (excitation wavelengths 561 and 780 nm (red – cells, cyan - NPs)) (b).

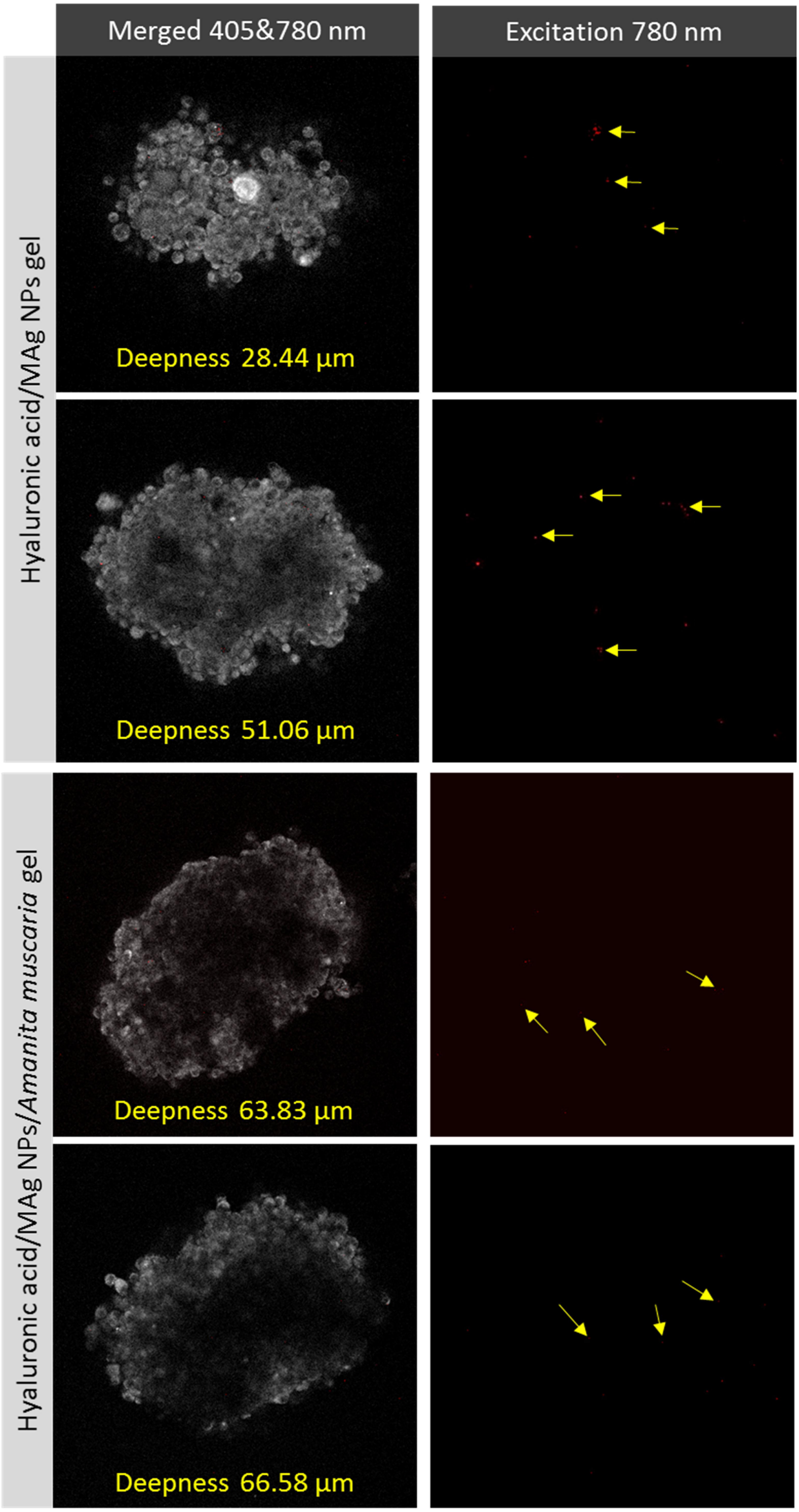

Fig. S13. Fluorescence images of HeLa spheroids (slices of 3D scans) at excitation wavelengths 405 and 780 nm showing the presence of NPs on different spheroids depth. Excitation wavelengths 405 nm caused auto fluorescence of cells (grey), and excitation 780 nm allowed to visualize NPs (red).

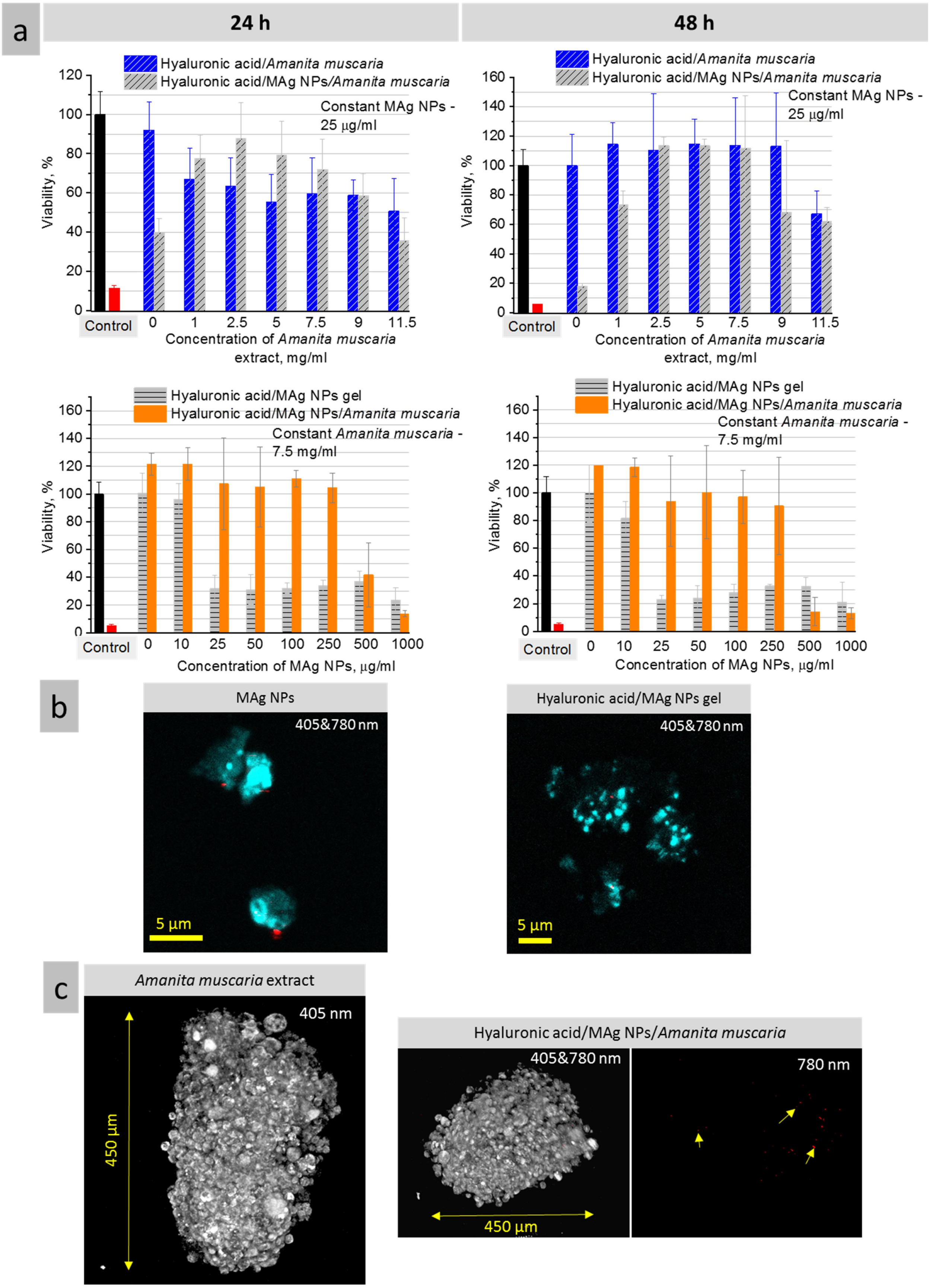

Fig. S14. Cytotoxicity study of gels with *Amanita muscaria* extract: viability of 2D HeLa cells after exposition with hyaluronic acid/*Amanita muscaria*, hyaluronic acid/MAg/*Amanita muscaria* and hyaluronic acid/MAg NPs (for comparison) gels (n=3, ±SD) (extract concentration was estimated by weighing of extract dry residue, drying temperature was 50 °C) (a); fluorescence images of 2D HeLa cells after 24 h of exposition with samples (excitation wavelengths 405 nm (autofluorescence of cells, cyan) and 780 nm (MAg NPs emission, red)), the arow points to the broken membrain of cell) (b); fluorescence 3D images of HeLa spheroids (representative images of 3 spheroids are shown), excitation wavelengths 405 nm (autofluorescence of cells, grey) and 780 nm (MAg NPs emission, red) (c).

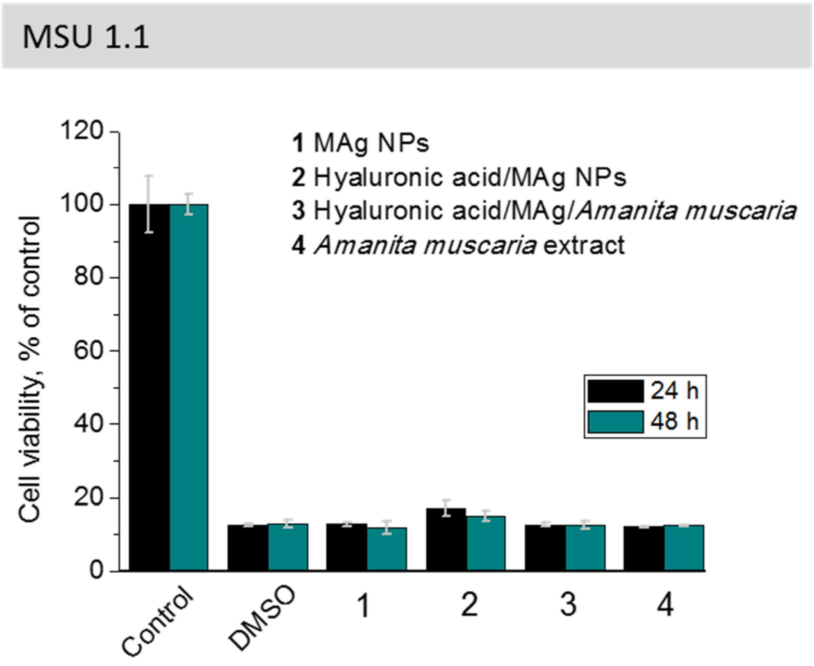

Fig. S15. Human fibroblast cells MSU-1.1 viability after interaction with MAg NPs, hyaluronic acid/MAg NPs and hyaluronic acid/MAg/*Amanita muscaria* gels, *Amanita muscaria* extract (concentration of MAg NPs - 25 µg/ml, *Amanita muscaria* – 7.5 mg/ml).
